# Comprehensive Analysis of Prognosis and Immune Landscapes Based on Lipid-Metabolism- and Ferroptosis-Associated Signature in Uterine Corpus Endometrial Carcinoma

**DOI:** 10.3390/diagnostics13050870

**Published:** 2023-02-24

**Authors:** Pusheng Yang, Jiawei Lu, Panpan Zhang, Shu Zhang

**Affiliations:** 1 Shanghai Key Laboratory of Gynecology Oncology, Department of Gynecology and Obstetrics, Renji Hospital, Shanghai Jiao Tong University School of Medicine, Shanghai 200127, China; 2 Department of Gastroenterology, Shanghai General Hospital, Shanghai Jiao Tong University School of Medicine, Shanghai 201620, China; 3 Shanghai Key Laboratory of Pancreatic Diseases, Shanghai General Hospital, Shanghai Jiao Tong University School of Medicine, Shanghai 201620, China

**Keywords:** lipid metabolism, ferroptosis, immunotherapy, prognostic marker, uterine corpus endometrial carcinoma

## Abstract

(1) Background: The effect of tumor immunotherapy is influenced by the immune microenvironment, and it is unclear how lipid metabolism and ferroptosis regulate the immune microenvironment of uterine corpus endometrial carcinoma (UCEC). (2) Methods: Genes associated with lipid metabolism and ferroptosis (LMRGs-FARs) were extracted from the MSigDB and FerrDb databases, respectively. Five hundred and forty-four UCEC samples were obtained from the TCGA database. The risk prognostic signature was constructed by consensus clustering, univariate cox, and LASSO analyses. The accuracy of the risk modes was assessed through receiver operating characteristic (ROC) curve, nomogram, calibration,, and C-index analyses. The relationship between the risk signature and immune microenvironment was detected by the ESTIMATE, EPIC, TIMER, xCELL, quan-TIseq, and TCIA databases. The function of a potential gene, PSAT1, was measured by in vitro experiments. (3) Results: A six-gene (CDKN1A, ESR1, PGR, CDKN2A, PSAT1, and RSAD2) risk signature based on MRGs-FARs was constructed and evaluated with high accuracy in UCEC. The signature was identified as an independent prognostic parameter and it divided the samples into high- and low-risk groups. The low-risk group was positively associated with good prognosis, high mutational status, upregulated immune infiltration status, high expression of CTLA4, GZMA and PDCD1, anti-PD-1 treatment sensitivity, and chemoresistance. (4) Conclusions: We constructed a risk prognostic model based on both lipid metabolism and ferroptosis and evaluated the relationship between the risk score and tumor immune microenvironment in UCEC. Our study has provided new ideas and potential targets for UCEC individualized diagnosis and immunotherapy.

## 1. Introduction

Uterine corpus endometrial carcinoma (UCEC) is one of the most common gynecologic malignancies, with an increasing incidence of about 1% per year [1]. Approximately 15% of UCEC patients are diagnosed at an advanced stage, and approximately 15–20% of patients will experience relapse after primary surgical treatment [2,3]. Although surgery, carboplatin/paclitaxel systemic chemotherapy, and hormone therapy are effective treatments, patients with advanced disease, recurrence, or drug resistance still have poor prognoses [4,5]. In recent years, it has been reported that patients with advanced endometrial cancer may benefit from immunotherapy. The main immunotherapy approaches include immune checkpoint inhibitors (ICIs), adoptive cell transfer (ACT), cancer vaccines, and lymphocyte-promoting cytokines. For example, dostarlimab, a drug that inhibits the programmed cell death 1 and programmed cell death ligand 1 pathway, can improve the prognosis of patients receiving platinum chemotherapy or progressive mismatch repair deficiency endometrial cancer [6]. However, the effect of immunotherapy is not ideal due to the complexity of the immune microenvironment and differences in the response to immunotherapy [7,8]. Therefore, it is vital to identify potential diagnostic and prognostic targets or risk signatures and to tailor individualized immunotherapy strategies for improving the outcomes of UCEC patients.

Obesity is an independent risk factor for UCEC [9]. Almost all UCEC patients with obesity have altered lipid metabolism [10]. Tan et al. built an 11 lipid metabolism gene (LMG) signature to reflect the prognosis of UCEC patients [11]. Lipids are susceptible to oxidation by oxygen free radicals. Overproduction and elimination failure of lipid peroxidation are the main reasons for the novel iron-dependent cell death ferroptosis [12,13,14]. Liu et al., Wang et al., and Wei et al. constructed a ferroptosis-related gene signature to predict the prognosis of UCEC patients [15,16,17]. Lipid synthesis, storage, and degradation processes can be regulated by ferroptosis [18,19]. Iron depletion leads to a large amount of lipid accumulation in breast cancer cells [20]. Iron accumulation is due to altered lipid metabolism associated with increased oxidative stress in myelodysplastic syndromes [21]. Ferroptosis is closely associated with lipid metabolism pathways [22,23]. Inhibiting β-oxidation can restore tumor cell sensitivity to ferroptosis [24]. Upregulating stearoyl CoA desaturase 1 (SCD1), the rate-limiting enzyme in fatty acid synthesis, increases the resistance of tumor cells to ferroptosis. Increasing evidence suggests that lipid metabolism and ferroptosis closely affect each other [25,26]. However, the interaction and shared role of ferroptosis and lipid metabolism in UCEC remains unclear.

In the present study, we aimed to construct a prognostic risk signature based on both lipid metabolism and ferroptosis to comprehensively analyze their combined effects on UCEC. We screened six risk genes (CDKN1A, ESR1, PGR, CDKN2A, PSAT1, and RSAD2) as reliable diagnostic and prognostic biomarkers and divided UCEC patients into high- and low-risk groups based on their risk score. Then, we estimated differences in immune score, immune infiltration, immune checkpoint, immunotherapy, and chemotherapy response between the high- and low-risk groups. The findings provide a new idea for individualized therapy strategies to improve the prognosis of UCEC patients.

## 2. Materials and Methods

### 2.1. Dataset Information

Sequencing RNA data (HTSeq-FPKM) and clinical information were obtained from The Cancer Genome Atlas (TCGA) database, and 579 cases were selected for study, including 544 UCEC samples and 35 normal samples. The detailed clinical information of the UCEC patients is shown in Table S1.

### 2.2. Extraction of Lipid-Metabolism-Related and Ferroptosis-Associated Genes

Lipid-metabolism-related genes (LMRGs) were collected from the Kyoto Encyclopedia of Genes and Genomes (KEGG) database and the Molecular Signatures Database (MSigDB), including the GSEA, HALLMARK, and REACTOME databases [27]. The detailed gene sets are shown in Table S2. A total of 1457 genes were selected for analyses after removing duplicate genes (Table S3). In addition, we downloaded 288 ferroptosis-associated genes (FAGs) from the FerrDb database (http://zhounan.org/ferrdb/legacy/index.html, accessed on 1 June 2022). After removing the replicates, 259 individual FAGs were used for further investigation.

### 2.3. Construction of the LMRG and FAR Prognostic Signature

The evaluation of the differentially expressed LMRGs (DE-LMRGs) was performed using the default settings for the “lmFit”, “eBayes”, and “topTable” functions in the “limma” R package. The screening criteria were *p* < 0.05, |Log2 Fold Change (FC)| > 1, and a false discovery rate (FDR) < 0.05. Then, univariate Cox regression analysis was applied to determine LMRGs with overall survival (OS) in UCEC by using the coxph function in the “survival” R package at *p* < 0.05. The molecular classification of DE-LMRGs in UCEC was analyzed by the “ConsensusClusterPlus” R package. Principal component analysis (PCA) was performed to identify the grouping ability of our model with the R package “stats”. Then, the FAGs interacted with the results of the consensus clustering approach, and the genes of interaction were selected for further study.

We performed univariate cox and least absolute shrinkage and selection operator (LASSO) analyses to identify significant prognostic genes based on both LMRGs and FARs with a threshold of *p* < 0.05. Then, a risk score signature was created by considering the estimated cox regression correlation coefficients and the expression values of the optimized LMRGs and FARs. The formula is risk score = Σi1expGenei*coeffi. According to the median value of the calculated risk scores from the TCGA-UCEC, the patients were divided into low- and high-risk groups. The prognostic ability and stability of the signature was measured by the Kaplan–Meier (K–M) analysis, multivariate Cox regression analysis, and receiver operating characteristic (ROC) curve with the “Survival” and “sevivalROC” R package (*p* < 0.05).

### 2.4. Functional Enrichment Analysis

To examine the distinction between the high- and low-risk group of our model, we further carried out gene set variation analysis (GSVA) using the “GSVA” function with method parameters (min.sz = 10, max.sz = 500, verbose = TRUE) of the “GSVA” R package, and conducted KEGG pathway analysis and Gene Ontology (GO) analysis via the “clusterProfiler (version 3.14.3)” R package (*p* < 0.05).

### 2.5. Tumor Mutational Burden (TMB) Analysis

We downloaded the somatic mutation data from TCGA. Using Perl, we calculated the TMB value of each sample and divided all samples into high- and low-TMB groups based on the median TMB [28]. Then, K–M analysis was used to assess survival differences between the groups. We also calculated the expression differences in TMB between the high- and low-risk groups and analyzed the relationship between TMB and the risk score (*p* < 0.05)

### 2.6. Immune Infiltration of the Prognostic Model

The CIBERSORT algorithm was utilized to evaluate the 22 types of immune fractions between the high- and low-risk groups, and the results were visualized with the “vioplot” R package. Then, we used the Tumor Immune Estimation Resource (TIMER) to evaluate correlations between expression of six model genes and the immune infiltration level of tumor-infiltrating immune cells. We also analyzed the relationship between innovative targeted therapy and risk prognostic models. The Wilcoxon test was used to detect expression of potential immune checkpoints between the high-risk and low-risk groups (*p* < 0.05). Furthermore, we downloaded clinical data from The Cancer Immunome Atlas (TCIA) to predict the response to immune checkpoint blockade (CTLA-4 and PD-1) in patients in the high- and low-risk groups by the immunophenoscore. In addition, according to the Genomics of Drug Sensitivity in Cancer (GDSC) database, the R package “pRRophetic” was used to measure the half-maximal inhibitory concentration (IC50) of chemotherapeutic drugs.

### 2.7. Cell Culture

The UCEC cell lines Ishikawa, HEC-1A, HEC-1B, and ECC-1 were obtained from the American Type Culture Collection (ATCC). The HEC-1A cell lines were cultured in McCoy’s 5A (Gibco, New York, NY, USA) supplemented with 10% fetal bovine serum (FBS, Biological Industries, Kibbutz Beit-Haemek, Israel) and 1% penicillin/streptomycin (P/S); the others were cultured in RPMI 1640 culture medium with 10% FBS and 1% P/S. All of the cells were cultured at 37 °C in a humidified incubator under 5% CO_2_.

### 2.8. Small Interfering RNA (siRNA) Transfection

The siRNA PSAT1 and scrambled control sequences were obtained from Gene Pharma (Shanghai, China). The details of the sequences are as follows: si-PSAT1-1: forward 5′-CAGUGUUGUUAGAGAUACAdTdT-3′, reverse 5′-UGUAUCUCUAACAACACUGdTdT-3′; si-PSAT1-2: forward 5′-GCUGUUCCAGACAACUAUAdTdT-3′, reverse 5′-UAUAGUUGUCUGGAACAGCdTdT-3′. siRNA transfection was carried out using Lipofectamine 2000 (Invitrogen, Carlsbad, CA, USA).

### 2.9. Quantitative Real-Time PCR (qRT–PCR)

Total RNA was extracted using TRIzol reagent (Sangon Biotech, Shanghai, China) after transfecting siRNA for 48 h, and reverse transcription was performed using PrimeScriptTM RT Reagent Kit (TAKARA, RR047A). QRT–PCR was conducted with the SYBR Green qPCR Supermix kit (Invitrogen). The primers used were purchased from Tsingke Biotechnology Co (Beijing, China), as follows: PSAT1 Forward 5′-ACTTCCTGTCCAAGCCAGTGGA-3′; PSAT1 Reverse 5′-CTGCACCTTGTATTCCAGGACC-3′; GAPDH Forward 5′-GGAGCGAGATCCCTCCAAAAT-3′; GAPDH Reverse 5′-GGCTGTTGTCATACTTCTCATGG-3′.

### 2.10. Western Blot Analysis

Total proteins were obtained from cells using PIPA buffer (New Cell & Molecular Biotech, Suzhou, China) at 72 h after siRNA transfection, separated by sodium dodecyl sulfate–polyacrylamide gel electrophoresis (SDS–PAGE), and transferred to polyvinylidene fluoride (PVDF) membranes (Millipore, New York, NY, USA). The membranes were blocked using 5% BSA for at least 1 h at room temperature and incubated with PSAT1 (10501-1-AP, Proteintech, Wuhan, China) or GAPDH (10494-1-AP, Proteintech) at 4 °C overnight. The next day, the membranes were incubated with secondary antibody (GB23303, Servicebio, Shanghai, China) for 1 h at room temperature, and bands were detected by chemiluminescence.

### 2.11. Cell Proliferation Assay

Cell proliferation was detected by the Cell Counting Kit-8 assay (CCK-8) and colony formation assay. For CCK-8, the cells were seeded into 96-well plates at a density of 2000 cells/well after 72 h of transfection. At the indicated time, CCK-8 solution (10 μL) was added to each well of the culture medium. Cell viability was measured using an automatic enzyme-linked immune detector after incubation for 1 h. For the colony formation assay, 1000 transfected cells were seeded into six-well plates for 10–14 days, and the culture medium was changed every three days. After staining with 0.1% crystal violet and photographing, cell colonies were statistically analyzed by the *t*-test.

### 2.12. Cell Migration and Invasion Assay

Cell migration and invasion were assessed using 24-well transwell chambers (8 μm; Millipore). In brief, a sample of 4 × 10^4^ cells suspended in 200 μL serum-free medium was seeded in the upper chamber, and the lower chamber contained 600 μL medium with 10% FBS. After 48 h, the chambers were fixed with 4% paraformaldehyde and stained with 0.1% crystal violet dye for 30 min. The upper chamber cells were wiped off and then photographed and counted under a microscope. For the invasion assays, Matrigel (BD, biocoat, #358248) was used to coat the upper chamber, after which the cells were seeded; the next step was the same as above.

### 2.13. Statistical Analysis

Bioinformatic statistical analyses were performed using R (v.3.6.1) software. Pearson correlation analysis was employed for correlation analysis between TMB and the risk model. All of the in vitro experiments were independently performed in triplicate and analyzed by the *t*-test. Data were analyzed using the IBM SPSS Statistics 22 and visualized in GraphPad Prism 9. The values were presented as the mean ± standard deviation (SD). *p* < 0.05 was considered statistically significant.

## 3. Results

### 3.1. Identification and Clustering of LMRGs

**Figure 1:**
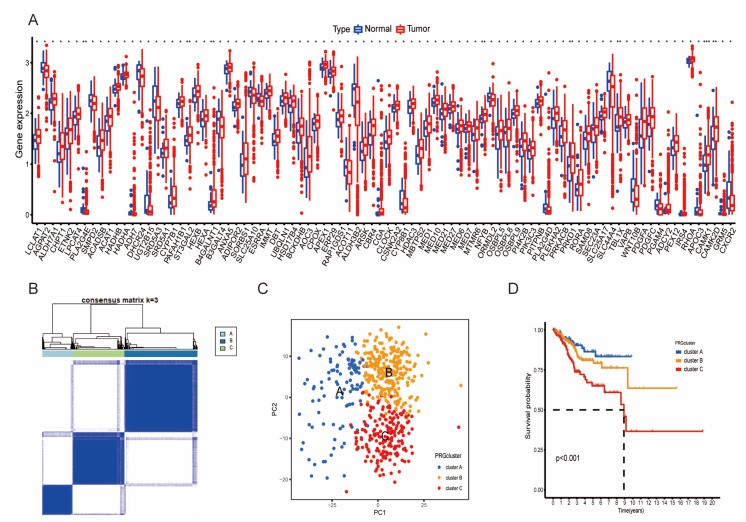
Identification and clustering of LMRGs: (**A**) expression of 88 DE-LMRGs between the UCEC samples and normal samples. * *p* < 0.05, ** *p* < 0.01, and *** *p* < 0.001. (**B**) Consensus clustering map of NMF clustering, and the optimal cluster number was three (k value = 3). (**C**) Principal component analysis (PCA) showed that the three clusters in the consensus clustering approach were robustly segregated. (**D**) Kaplan–Meier curve survival (K–M) analysis of patients in the three clusters, and patients in Cluster A had a better prognosis. *p* < 0.001.

A brief workflow of this research is presented in Figure S1. We screened 1457 LMRGs for differential expression analysis and identified 88 differentially expressed LMRGs (DE-LMRGs) with the “limma” R package based on 544 UCEC samples and 35 normal samples from TCGA. The boxplot of the expression patterns of the 88 DE-LMRGs is shown in Figure 1A. KEGG analysis and GO analysis showed that these significant genes mainly participate in lipid metabolic processes (Figure S2A,B). Then, univariate cox hazards regression and Kaplan–Meier (K–M) analyses were utilized to screen out prognostic LMRGs based on TCGA, and we obtained six risk genes and three protective genes for survival (Figure S2C,D). The consensus clustering approach was used to divide the UCEC samples with the non-negative matrix factorization (NMF) algorithm. Based on LMRGs expression, the optimal clustering stability was confirmed when K = 3 (Figure 1B and Figure S2E,F). We also performed principal component analysis (PCA), which showed the good grouping ability of our clustering (Figure 1C). Therefore, all of the UCEC samples were divided into three clusters, and the heatmap showed lower expression for the DE-LMRG genes in Cluster A (Figure S2G). Moreover, K–M analysis indicated a significant difference in OS among the three subgroups, with the patients in Cluster A having the best prognosis (Figure 1D, *p* < 0.05). By further analyzing the clinical characteristics among the three clusters, we found that patients in Cluster C had an older age and a higher grade and stage (Figure S2H–J, *p* < 0.05).

### 3.2. Signature Construction Based on LMRGs and FAGs

**Figure 2:**
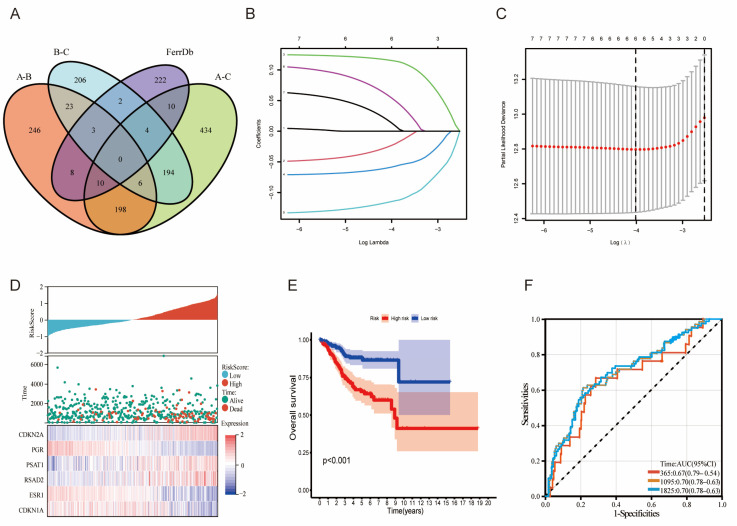
Constructing the LMR-FAG-related risk score. (**A**) Venn diagram showing lipid-me tabolism-related and ferroptosis-associated genes (LMR-FAGs). (**B**,**C**) LASSO coefficient profiles and cross-validation were used to identify LMR-FAG-related genes. (**D**) Distributions of risk scores, survival status, and expression levels of six prognostic genes in UCEC. (**E**) The K-M survival analysis demonstrated that patients in the low-risk group had a better prognosis. *p* < 0.001. (**F**) Time-dependent ROC curve and AUC of the prognostic signature in UCEC patients from TCGA.

Differentially expressed genes among the three clusters were obtained from consensus clustering analysis and intersected with FAGs. Then, we obtained both lipid metabolism-related and ferroptosis-associated genes (LMG-FAGs) (Figure 2A). We performed overall-survival-based univariate regression analysis on the lipid-metabolism-related and ferroptosis-associated genes (LMG-FAGs) obtained through consensus clustering analysis. This approach revealed 211 LMG-FAGs associated with the prognosis of endometrial cancer, and we classified them into 87 risk genes and 124 protective genes according to the hazard ratio (HR) and *p* value (Table S4, *p* < 0.05). To avoid overfitting and bias, the results of univariate regression analysis were subjected to LASSO regression analysis using the “glmnet” R package, and the accuracy of the model was tested by cross-validation (Figure 2B,C). Hence, a six-gene prognostic risk model was established by the following formula: risk score = [CDKN1A expression × (−0.02353)] + [CDKN2A expression × (0.11554)] + [ESR1 expression × (−0.05874)] + [PGR expression × (−0.11493)] + [PSAT1 expression × (0.05505)] + [RSAD2 expression × (0.01431)]. We analyzed the relationship between different risk scores and patient follow-up times, events, and expression changes of individual genes, and it was observed that with an increase in the risk score, the survival rate of patients decreased significantly. CDKN1A, ESR1, and PGR were found to be protective factors that showed downregulated expression with increased risk scores; CDKN2A, PSAT1, and RSAD2 showed the opposite result (Figure 2D *p* < 0.05). Furthermore, we detected expression levels and performed multivariate Cox regression and K-M survival analyses on the six independent prognostic genes. The results indicated that high expression of CDKN1A, ESR1, and PGR was related to better prognosis, whereas high expression of CDKN2A, PSAT1, and RSAD2 was not (Figure S3A–C, *p* < 0.05). According to the median cut-off value of the risk score, the high- and low-risk groups were established to differentiate the UCEC patients in TCGA, and the high-risk patients had a worse prognosis than the low-risk patients (Figure 2E, *p* < 0.05). Then, time-dependent ROC analysis was applied to evaluate the prediction capacity of the signature, with an area under the receiver operating characteristic curve (AUC) of 0.67, 0.70, and 0.70 at 365, 1905, and 1825 days, respectively (Figure 2F, *p* < 0.05).

### 3.3. Prognosis and Validation of the LMRG- and FAG-Based Signature

**Figure 3:**
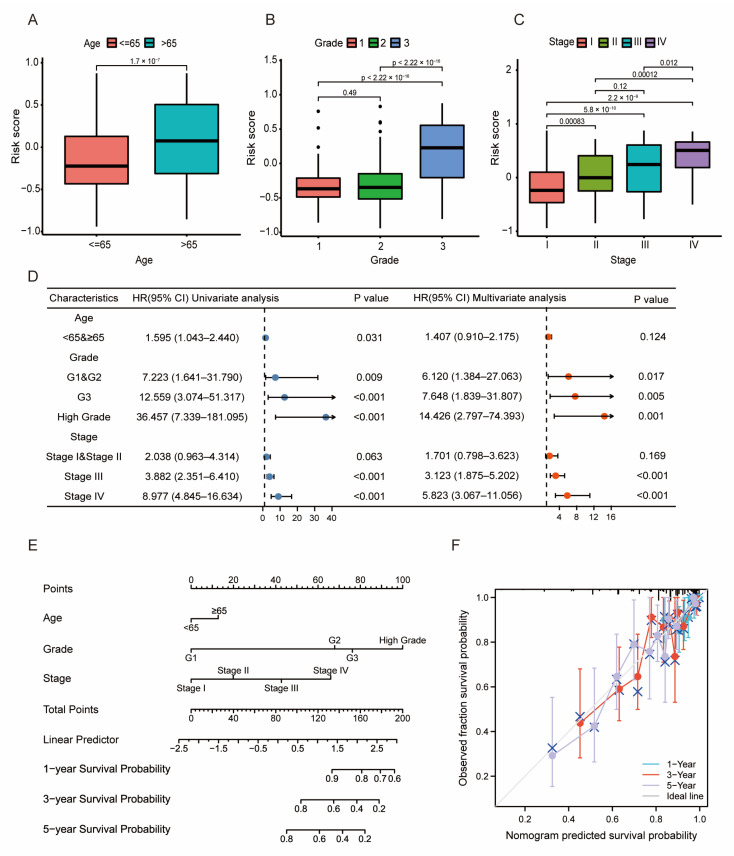
Establishment of a predictive nomogram. (**A**–**C**). The pathological characteristics age, grade, and stage in the high- and low-risk groups. Patients in the high-risk group had an older age and higher grade and stage. (**D**) Univariate and multivariate Cox regression analyses of the signature and clinical characteristics. (**E**) Nomogram to predict 1-year, 3-year, and 5-year overall survival times. (**F**) Calibration curve to assess the accuracy of the nomogram. The C-index was 0.767 (0.741–0.793).

To assess the accuracy of the model, we evaluated the performance of this signature with regard to pathological features (age, grade, and stage). The results indicated that high risk was significantly associated with older age and higher grade and stage (Figure 3A–C, *p* < 0.05). Then, the pathological features were added for univariate and multivariate cox regression, and the forest plot showed that age, grade, and stage were still independent prognostic factors, which means that the signature had high accuracy (Figure 3D, *p* < 0.05). In addition, we built a nomogram to predict the 1-year, 3-year, and 5-year survival probability of UCEC patients based on all of the above prognostic elements (Figure 3E, *p* < 0.05), and the calibration plot showed a C-index of 0.767 (0.741–0.793), indicating that the nomogram had good predictive ability (Figure 3F, *p* < 0.05).

### 3.4. DEG and Functional Enrichment Analyses of the Signature

**Figure 4:**
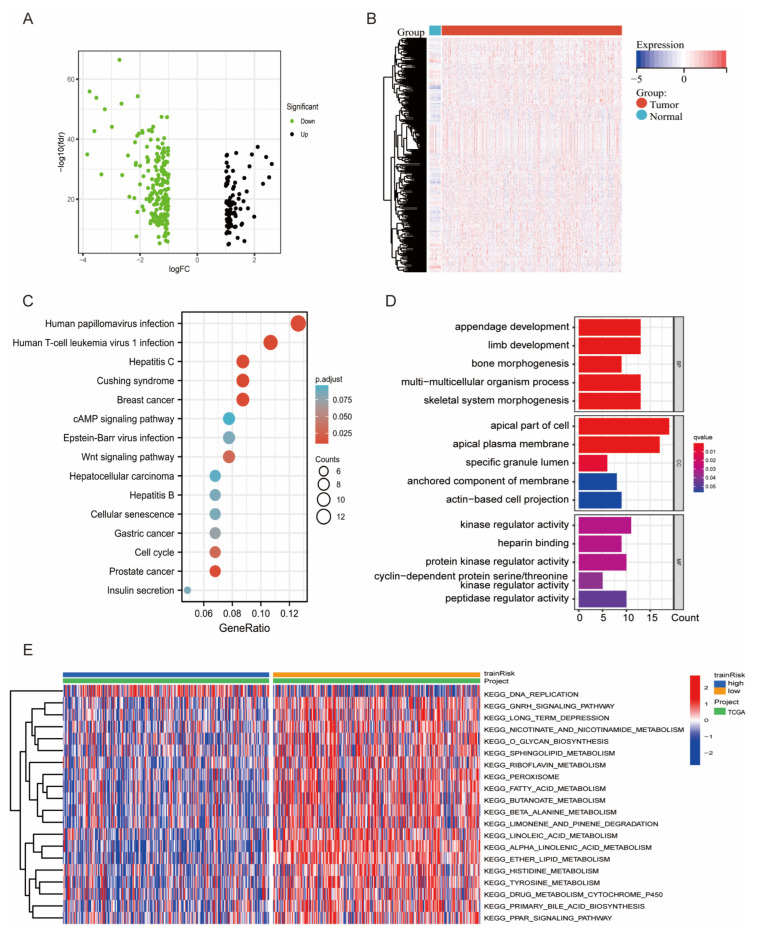
DEGs and functional enrichment of the signature. (**A**,**B**) The volcano plot and heatmap show the details of different genes between the high- and low-risk groups. (**C**,**D**) KEGG and GO functional enrichment analysis in terms of differentially expressed genes. (**E**) Gene set variation analysis between high- and low-risk patients based on TCGA-UCEC.

To investigate the relationship between the six genes in the risk model, we constructed a protein–protein interaction (PPI) network (Figure S4A) and analyzed the correlations (Figure S4B). The results showed that PSAT1 and RSAD2 were more independent and less associated with other genes. Next, a volcano plot and heatmap showed the DEGs between the two risk groups; 81 genes were upregulated and 195 genes were downregulated (Figure 4A,B). The PPI network of the DEGs is depicted in Figure S4C. To reveal the underlying biological characteristics associated with the risk scores, KEGG and GO analyses were performed based on DEGs between the high- and low-risk groups. The results indicated that pathways such as kinase and peptidase regulation, apparatus morphogenesis, cell cycle regulation, viral infection, and antiviral innate immune response were highly enriched (Figure 4C,D, *p* < 0.05). In addition, we performed GSVA to probe differences in pathways between the two risk groups. As illustrated in the heatmap in Figure 4E, pathways related to lipid metabolism and ferroptosis, such as “tyrosine metabolism”, “fatty acid metabolism”, “alpha linolenic acid metabolism”, and “DNA replication”, were significantly enriched (*p* < 0.05).

### 3.5. Relationship between the Tumor Mutational Burden (TMB) and the Risk Model

**Figure 5:**
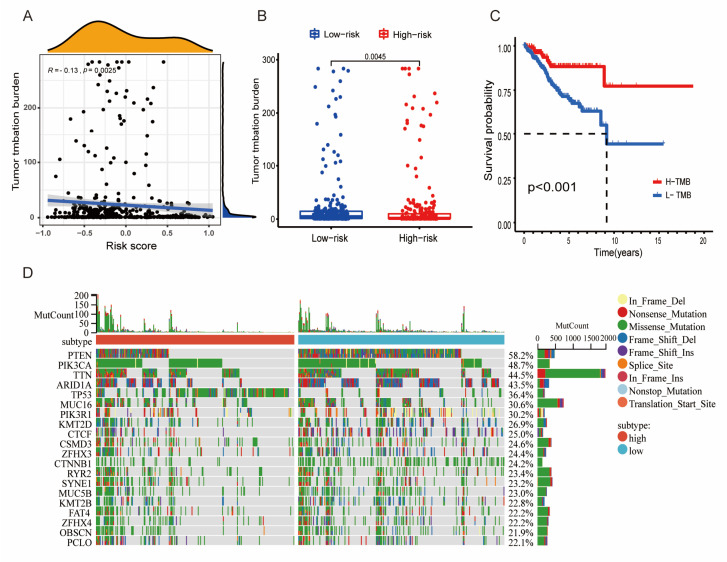
Relationship between the tumor mutational burden (TMB) and the risk model. (**A**) Relationship between TMB and risk score. TMB was negatively associated with risk score. (R = −0.13, *p* < 0.05). (**B**) The result showed that patients in high-risk group had lower TMB levels. *p* < 0.05 (**C**) The K-M analysis presents the difference in overall survival between the low- and high-TMB groups. In addition, patients with high TMB had a better prognosis, *p* < 0.05. (**D**) The waterfall plot shows the mutation information of the top 20 genes in each UCEC sample.

TMB, the somatic coding errors, is generally considered high when >10 or >16 mutations/megabase DNA are present [28]. Recently, TMB is thought to be closely related to the survival prognosis of tumor patient [29]. To examine in more depth how well the risk-prognosis model predicts tumor development, we investigated its relationship with TMB. First, correlation analysis showed that the TMB level had a negative association with the LMRG-FAG risk score (Figure 5A, *p* < 0.05), and the high-risk group showed lower TMB levels (Figure 5B, *p* < 0.05). We also investigated the survival of patients with different TMB statuses by K-M analysis, and the results demonstrated that the patients in the low-TMB group had poor prognostic outcomes (Figure 5C, *p* < 0.05). In addition, mutation information of the genes in the low- and high-TMB groups was explored using a waterfall chart, and PTEN (58.2%), PIK3CA (48.7%), TTN (44.5%), ARID1A (43.5%), and TP53 (36.4%) were the top five mutated genes (Figure 5D). We further studied and classified the mutation information, variant type, and SNV class, and the results demonstrated that missense mutations, single nucleotide polymorphism (SNP), and C > T accounted for the largest proportion (Figure S5A–C). The number of altered bases in each sample and the mutation types in different colors are shown in Figure S5D,E; mutation information for the six risk genes [PGR (37%), ESR1 (33%), RSAD2 (27%), PSAT1 (18%), CDKN1A (14%), and CDKN2A (5%)] is provided in Figure S5F. Recently, multiple pieces of research have illustrated that TMB is closely associated with tumor immune cell infiltration and affects the efficacy of immunotherapy [30,31]. Therefore, we evaluated the value of TBM in the complexity of the tumor immune microenvironment. We discovered that most immune cells had a positive correlation with the TMB level, especially T cells CD8+, T cells CD4+, and B cells (Figure S5G). In addition, T cells CD8+, T cells CD4+ memory activated, T cells CD4+ memory resting, and T cells regulatory had higher expression in the high-TMB group compared to the low-TMB group (Figure S5F, *p* < 0.05), suggesting that TMB may have an effect on the immune response.

### 3.6. Immune Infiltration Associated with the LMRG-FAG-Based Signature

**Figure 6:**
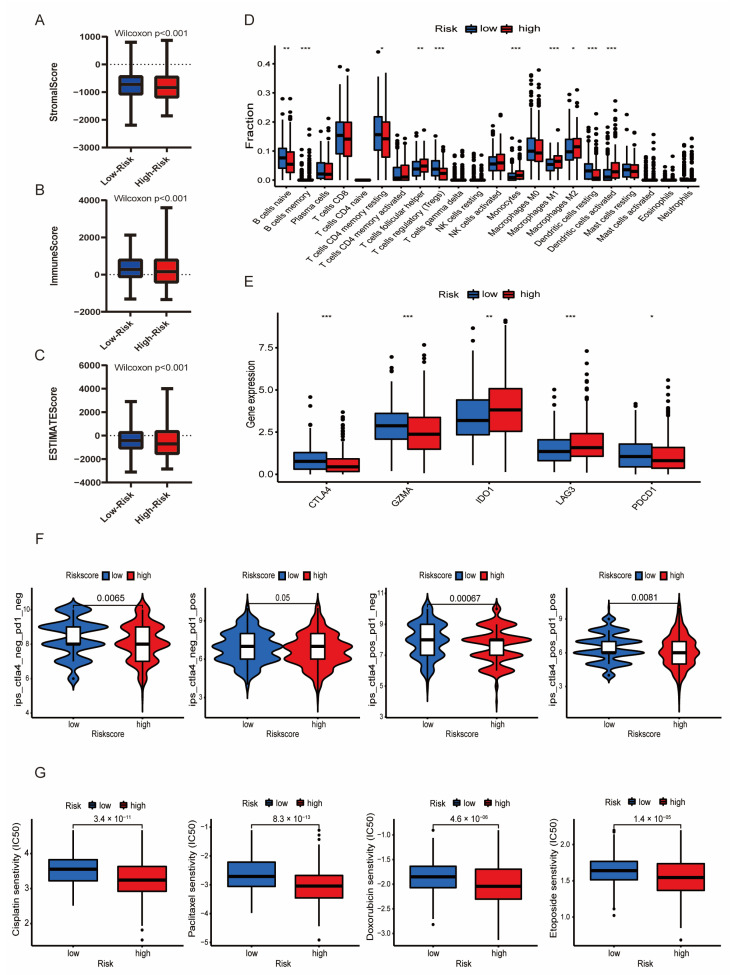
Immune landscape associated with the LMRG- and FAG-based signature. (**A**–**C**) Differences in stromal score, immune score, and ESTIMATE score between the high- and low-risk groups. (Wilcoxon, *p* < 0.05). (**D**) The difference in 22 immune infiltrating cells in the TCGA-UCEC samples between the high- and low-risk groups was analyzed by the Wilcoxon test. (**E**) The connection between immune checkpoint molecules (CTLA4, GZMA, IDO1, LAG3, and PDCD1) and risk scores. (**F**) Prediction of immunotherapy response to anti-PD-1 and anti-CTLA4 in patients in different risk groups. (**G**) Estimated IC50 values of four typical immunotherapy drugs (cisplatin, paclitaxel, doxorubicin, and etoposide) between the low- and high-risk groups. * *p* < 0.05, ** *p* < 0.01, and *** *p* < 0.001.

Recent studies have shown that lipid metabolism and ferroptosis are important components of the tumor microenvironment and are strongly associated with tumor immune activities [32,33,34,35]. We first used ESTIMATE to determine the relationship of tumor immune infiltration between the two risk groups. The stromal, immune score, and ESTIMATE score were significantly downregulated in the high-risk group (Figure 6A–C, Wilcoxon *p* < 0.05). Then, the CIBERSORT algorithm was applied to detect the composition of the 22 immune cells in UCEC patients (Figure S6A). A boxplot demonstrated that the difference in the distribution of the 10 immune-infiltrating cells between the two risk groups was significant. The naive B cells, memory B cells, resting CD4 memory T cells, regulatory T cells (Tregs), and resting dendritic cells had low expression in the high-risk group compared to the low-risk group. Meanwhile, the follicular helper T cells, monocytes, M1 macrophages, activated dendritic cells, and M2 macrophages were significantly upregulated in the high-risk group compared to the low-risk group (Figure 6D, *p* < 0.05). We also analyzed immune infiltration using the EPIC, TIMER, xCELL, and quanTIseq databases, which fully confirmed the six-gene prognostic risk signature to be closely related to immune activity (Figure S6B–E). In addition, the TIMER database was utilized to assess the relationship between the six risk genes and tumor-infiltrating immune cells. The results showed that only RSAD2 correlated positively with B cells (cor = 0.1858, *p* = 0.0015); except for RSAD2, the other genes were significantly associated with CD8+ T cells (Figure S7, *p* < 0.05).

### 3.7. Immunotherapy and Chemotherapy in Different Risk Groups

Recently, immune checkpoints have been identified as key targets of immunotherapy, and immune checkpoint inhibitors (ICIs) are regarded as an effective therapeutic strategy for patients with advanced disease [36,37]. Therefore, we identified potential relationships between the expression of immune checkpoint molecules and our risk model. The results showed that IDO1 and LAG3 expression was significantly increased in the high-risk group compared with the low-risk group, while the expression of CTLA4, GZMA and PDCD1 was obviously decreased in the high-risk group compared with the low-risk group (Figure 6E, *p* < 0.05). Then, we conducted immunophenoscore (IPS) analysis to predict immunotherapy response. As shown in Figure 6F, low-risk patients were more sensitive to anti-PD-1 therapy (*p* < 0.05), suggesting that immunotherapy of blocking CTLA-4 and PDCD1 may be more beneficial for patients in the low-risk group. Since chemotherapy is the main treatment for advanced and recurrent UCEC, we evaluated the response of chemotherapeutics to UCEC patients using the pRRophetic algorithm based on our signature and found that the estimated IC_50_ of typical chemotherapy drugs (cisplatin, paclitaxel, doxorubicin, and etoposide, etc.) were significantly higher in the low-risk group (Figure 6G, *p* < 0.05). For the other 40 chemotherapy and small molecule drugs, such as lenalidomide, gefitinib, AMG.706, and JNK inhibitor VIII, patients in the high-risk group were identified as being more sensitive (Figure S8, *p* < 0.05). Thus, we indicated that patients with low risk scores were more resistant to chemotherapy than those with high risk scores, but they were more sensitive to anti-PD-1 therapy. In addition, patients in the high-risk group were better suited for chemotherapy. These results may have important implications for individualized immunotherapy in patients with advanced UCEC.

### 3.8. In Vitro Function of the Risk Gene PSAT1 in UCEC Cells

**Figure 7:**
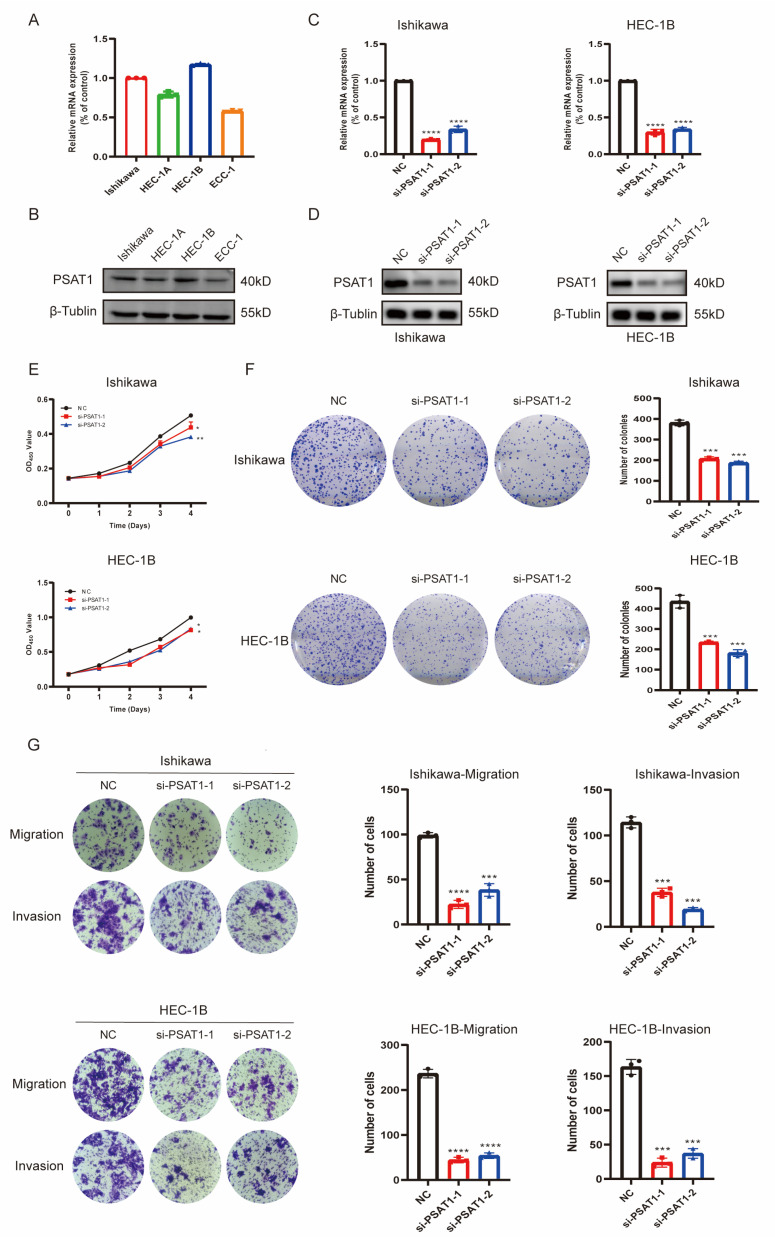
The risk gene PSAT1 promotes UCEC cells’ proliferation, migration, and invasion. (**A**,**B**) mRNA and protein levels of PSAT1 were measured by qPCR and Western blotting in four UCEC cell lines. (**C**,**D**) The efficiency of the knockdown of PSAT1 in Ishikawa and HEC-1B cells were measured by qPCR and Western blotting. (**E**,**F**) CCK-8 and colony formation assays showed that the proliferation ability of UCEC cells was decreased after PSAT1 knockdown. (**G**) The transwell assay showed that the migration and invasion capacities of UCEC cell lines were inhibited after PSAT1 knockdown. * *p* < 0.05, ** *p* < 0.01, *** *p* < 0.001, **** *p* < 0.0001.

To further validate the ability of risk signatures to predict prognosis, we investigated protein expression of the six risk genes between normal and UCEC tissues with the CPTAC and HPA (Human Protein Atlas) databases (Figure S9A,B, *p* < 0.05), and the results corresponded with previous analysis. Combined with prognostic analysis and literature searches, we selected PSAT1 for further in vitro functional assays. We identified the mRNA and protein expression of PSAT1 in four UCEC cell lines (Ishikawa, HEC-1A, HEC-1B, and ECC1), and Ishikawa and HEC-1B cells were selected for subsequent studies (Figure 7A,B). Next, we knocked down PSAT1 with siRNA, and the efficiency was verified by qPCR (Figure 7C, *p* < 0.05) and Western blot analysis (Figure 7D). CCK-8 and colony formation assays showed that knockdown of PSAT1 significantly suppressed the proliferation of Ishikawa and HEC-1B cells (Figure 7E,F, *p* < 0.05). In addition, the migration and invasion of the two cell lines were also apparently inhibited after PSAT1 knockdown, as determined by transwell assays (Figure 7G). These results demonstrate that the risk gene PSAT1 significantly promotes progression of UCEC and may affect the prognosis of UCEC patients.

## 4. Discussion

UCEC is one of the most lethal gynecological malignancies. Although many studies over the past decades have sought to improve treatment efficacy, patients with advanced and recurrent disease still have poor prognosis [38]. With the rise and application of immunotherapy, it is insufficient to estimate the prognosis of UCEC patients based on traditional clinicopathological stage [39]. Therefore, our study included the tumor immune microenvironment and immunotherapy in UCEC based on both lipid metabolism and ferroptosis to select more effective prognostic targets and guide individualized treatment of patients.

Previous studies have established prognostic models of lipid metabolism or ferroptosis in UCEC [11,15,16,17]. However, they only took a single influencing factor into account, and the complex tumor microenvironment was not considered. In our study, we comprehensively considered the interrelationship between lipid metabolism and ferroptosis, based on which a prognostic model of six genes was constructed. We deeply explored the relationship between the model risk score and the tumor immune microenvironment. We found that infiltration of B cells, T cells, and NK cells and expression of the immune checkpoints (CTLA4, GZMA, and PDCD1), as well as sensitivity and chemotherapy resistance to anti-PD-1 treatment in UCEC patients were closely related to the risk scores of the prognostic model. Moreover, in vitro experiments demonstrated that one of the potential targets, PSAT1, promoted the proliferation, migration, and invasion of UCEC cells. Our experiments provide new ideas and a basis for individualized immunotherapy for UCEC patients and provide a potential target for UCEC therapy.

In the present study, we obtained genes associated with both lipid metabolism and ferroptosis by consensus clustering analysis. After LASSO Cox regression, we constructed a prognostic signature containing six risk genes (CDKN1A, ESR1, PGR, CDKN2A, PSAT1, and RSAD2) based on LMG-FAGs. K-M survival analysis, ROC curves, a nomogram, and calibration identified that the signature had high predictive ability. Estrogen receptor 1 (ESR1) and a progesterone receptor (PGR) were reported to participate in lipid metabolism by encoding estrogen or steroid receptors to promote tumor progression [40,41,42]. Cyclin-dependent kinase inhibitors 1A and 2A (CDKN1A and CDKN2A) have been identified as ferroptosis-related genes in recent studies and can be regarded as biomarkers that influence the tumor microenvironment [43,44,45]. Radical s-adenosyl methionine domain containing 2 (RSAD2) is an interferon-stimulated gene that exerts antiviral effects by dysregulating cellular lipid metabolism [46,47]. Phosphoserine aminotransferase 1 (PSAT1) has been reported to affect the progression of various cancers by participating in lipid metabolism processes [48,49,50]. In conclusion, the six-gene prognostic model showed a significant correlation with lipid metabolism or ferroptosis. In our study, these six genes were used for risk scoring, and each UCEC patient was categorized into two risk groups according to the risk score. We then explored the pathological features of the risk signature, with the high-risk group being related to older age and higher grade and stage. We also found that knockdown of PSAT1 inhibited the proliferation, migration, and invasion of UCEC cells, enhancing the reliability of our model.

Subsequently, we comprehensively analyzed the impact of the risk signature on UCEC. A total of 276 genes were identified to be closely related to the risk score. GO, KEGG, and GSVA analyses based on the signature demonstrated that pathways associated with lipid metabolism and ferroptosis were significantly enriched, which also confirmed the accuracy of our signature. TMB is reported to correlate highly with tumor progression; for example, gastrointestinal tumor patients with low TMB have lower objective response rates and shorter progression-free survival [51], and high TMB is a poor prognostic factor for non-small cell lung cancer [52]. We found that TMB levels had a negative relationship with the LMRG-FAG risk model, which means that patients with low risk and high a mutational burden have a better prognosis in UCEC.

Because surgery and chemoradiotherapy have limited effects in patients with advanced and recurrent UCEC and traditional pathological staging has an insufficient ability to estimate prognosis, we focused on the relationship of the LMRG-FAG-based risk model with immunotherapy. Stromal, immune, and ESTIMATE scores were significantly downregulated in the high-risk group, indicating that lipid metabolism and ferroptosis are significantly associated with the immune status of UCEC. CIBERSORT algorithm analysis showed that the distribution of 10 immune cells varied between the high- and low-risk groups, with antitumor cells (B cells, T cell CD8, and monocytes, etc.) present at higher abundance in the low-risk group. According to the results, we suggest that the risk score is associated with immune infiltration and immune status in UCEC.

Adverse T cell regulatory pathways tend to be overactive when cancer occurs. CTLA-4 inhibits the immune response at the early stage of T cell induction, and PDCD1 prevents T cell function in peripheral tissues in the later stages [53,54]. Recently, immune checkpoint blockade, one of the major immunotherapy methods, has proven to be an effective strategy for enhancing the effector activity and clinical impact of anti-tumor T cells [55]. Among the ICIs, blocking CTLA-4 and PDCD1 are the two most eminent approaches. CTLA-4 and PDCD1 blockade could induce tumor immunity by improving effector T cell activity or consuming Treg [56]. In 2011, Ipilimumab, a CTLA-4 inhibitor, was approved for melanoma [57]. In 2017, the PDCD1 inhibitor pembrolizumab was approved for UCEC patients with microsatellite instability, and half of the patients benefited from it [58]. Since the predictive value of immune checkpoints has been demonstrated in a variety of human malignancies, we then explored immune checkpoint expression between the two risk groups to guide individualized immunotherapy for UCEC patients. The expression of CTLA4, GZMA and PDCD1 was significantly upregulated in patients with low risk scores, and IDO1 and LAG3 were increased in the high-risk group. Therefore, we indicated that specially blocking CTLA-4 and PDCD1 immunotherapy would be more effective for patients in the low-risk group. Meanwhile, we detected the difference in sensitivity to PD-1 and CTLA-4 inhibitors, and the results indicated that low-risk patients were more sensitive to anti-PD-1 therapy, meaning that immunotarget therapy was more effective in low-risk patients. Accordingly, our risk signature has a certain guiding role in the anti-PD-1 immunotherapy of UCEC patients. Interestingly, high-risk patients were more sensitive to traditional chemotherapeutic agents and small molecule inhibitors such as cisplatin, paclitaxel, AMG.706, and ABT.888. Hence, patients in the high-risk group were more likely to benefit from chemotherapy and our signature can be used to guide personalized treatment of UCEC patients.

However, it is undeniable that our study also has some limitations. First, the study data were obtained from only TCGA, and we did not verify the accuracy of our model with more cohorts. Second, immunotherapy and chemosensitivity analyses were only derived from the transcriptome, and we still need to obtain more prospective experimental data to support the findings. Finally, as a potential therapeutic target, the molecular mechanism underlying the risk-related gene PSTA1 needs to be further clarified.

## 5. Conclusions

Consequently, we constructed a risk prognostic model based on both lipid metabolism and ferroptosis to deeply analyze the relationship between lipid metabolism, ferroptosis and gene mutation, immune infiltration, immunotherapy, and chemotherapy in UCEC patients and provided potential biomolecules and a preliminary basis for individualized treatment of patients.

## Data Availability

Publicly available datasets were analyzed in this study. These data can be found here: https://www.cancer.gov/, accessed on 1 June 2022 (TCGA); https://www.ncbi.nlm.nih.gov/geo/, accessed on 1 June 2022 (GEO); http://zhounan.org/ferrdb/legacy/index.html, accessed on 1 June 2022 (FerrDb); https://ngdc.cncb.ac.cn/, accessed on 1 June 2022 (MSigDB).

## Supplementary Materials

The following supporting information can be downloaded at: https://www.mdpi.com/article/10.3390/diagnostics13050870/s1.
