# Supplementary material for: Comprehensive Analysis of Prognosis and Immune Landscapes Based on Lipid-Metabolism- and Ferroptosis-Associated Signature in Uterine Corpus Endometrial Carcinoma
Source: Diagnostics (Basel). 2023 Feb 24;13(5):870. doi: 10.3390/diagnostics13050870 (PMC10000778; doi:10.3390/diagnostics13050870)
Supplement: Supplementary file 1 [file diagnostics-13-00870-s001.zip › Supplementary Figures.pdf]

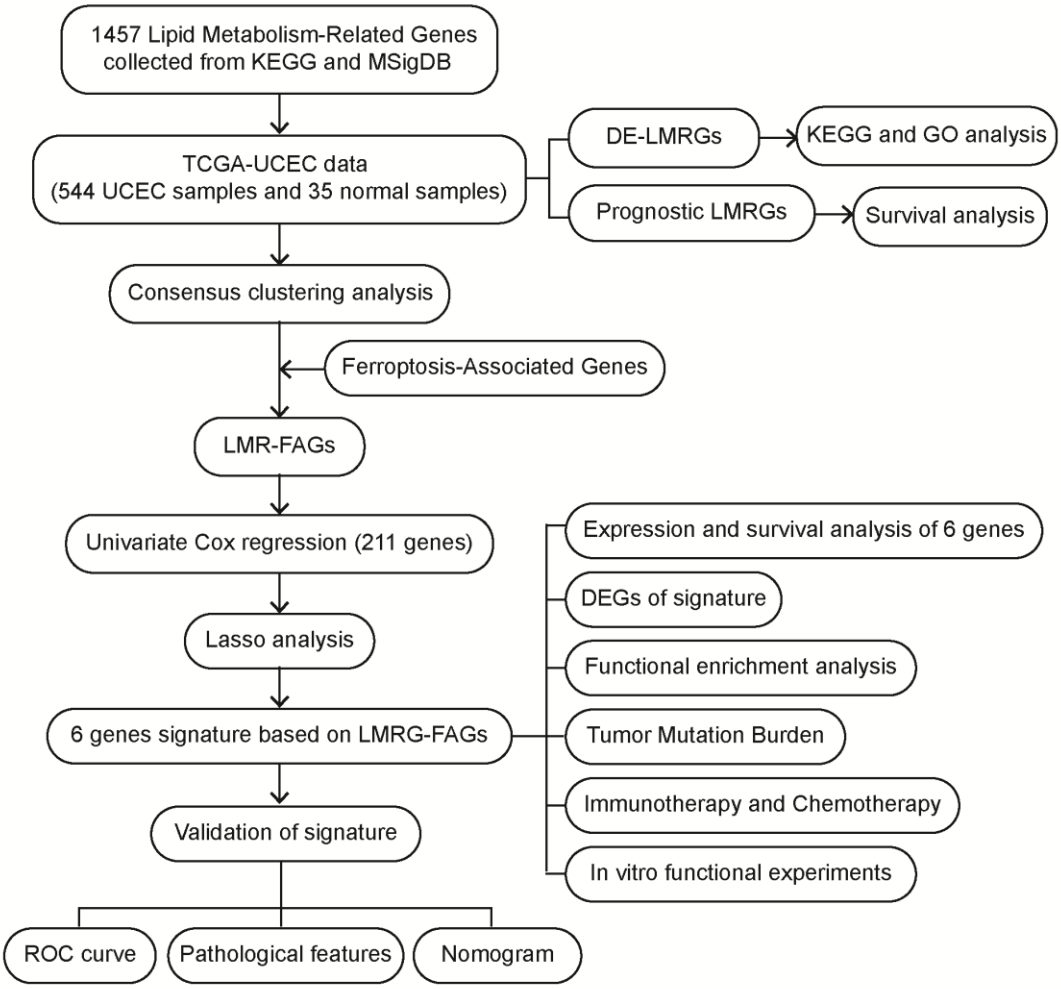

Figure S1. The brief workflow of this research.

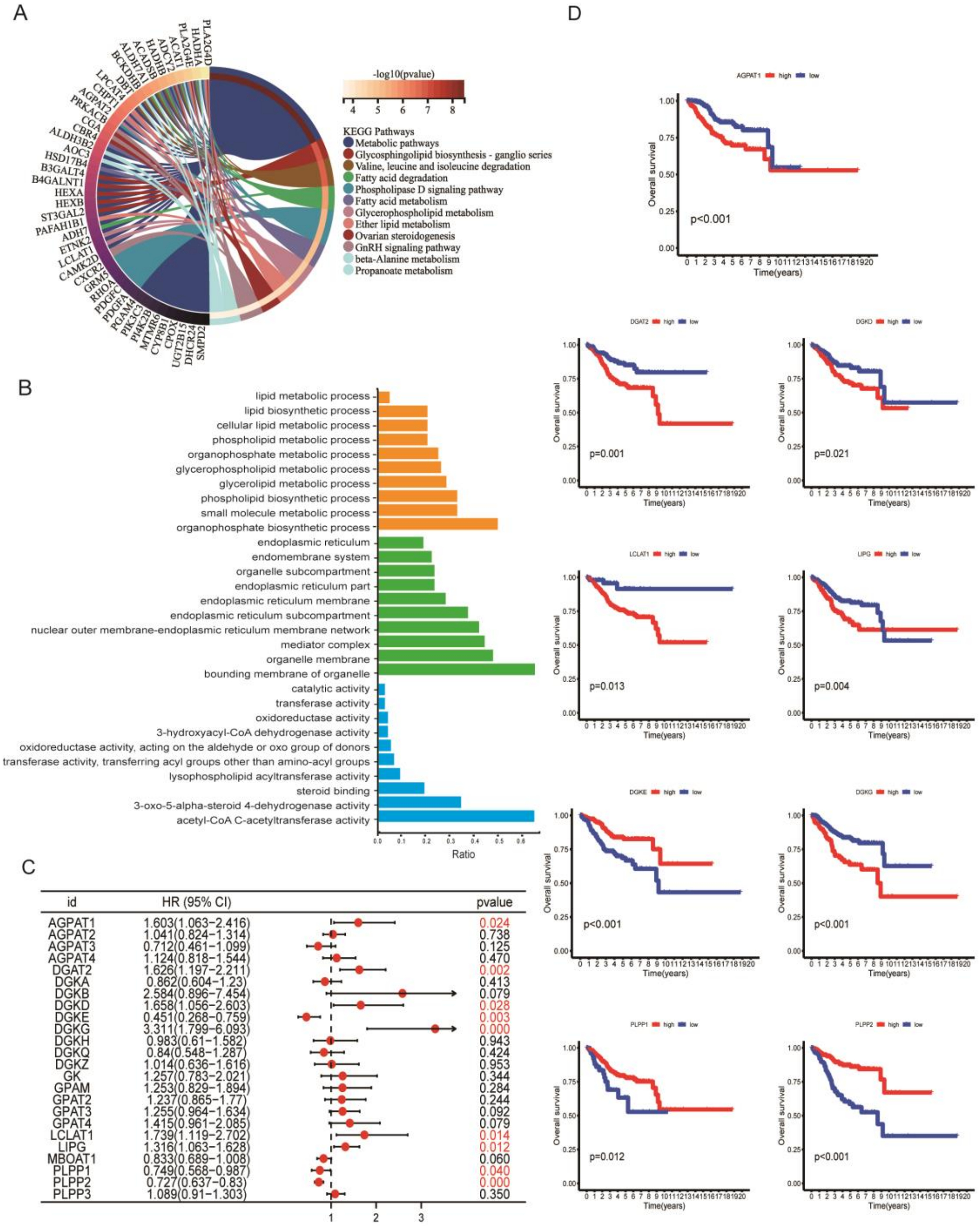
A
-log10(pvalue)
4 5 6 7 8
KEGG Pathways
Metabolic pathways
Glycosphingolipid biosynthesis - ganglio series
Valine, leucine and isoleucine degradation
Fatty acid degradation
Phospholipase D signaling pathway
Fatty acid metabolism
Glycerophospholipid metabolism
Ether lipid metabolism
Ovarian steroidogenesis
GnRH signaling pathway
beta-Alanine metabolism
Propanoate metabolism
B
lipid metabolic process
lipid biosynthetic process
cellular lipid metabolic process
phospholipid metabolic process
organophosphate metabolic process
glycerophospholipid metabolic process
glycerolipid metabolic process
phospholipid biosynthetic process
small molecule metabolic process
organophosphate biosynthetic process
endoplasmic reticulum
endomembrane system
organelle subcompartment
endoplasmic reticulum part
endoplasmic reticulum membrane
endoplasmic reticulum subcompartment
nuclear outer membrane-endoplasmic reticulum membrane network
mediator complex
organelle membrane
bounding membrane of organelle
catalytic activity
transferase activity
oxidoreductase activity
3-hydroxyacyl-CoA dehydrogenase activity
oxidoreductase activity, acting on the aldehyde or oxo group of donors
transferase activity, transferring acyl groups other than amino-acyl groups
lysophospholipid acyltransferase activity
steroid binding
3-oxo-5-alpha-steroid 4-dehydrogenase activity
acetyl-CoA C-acetyltransferase activity
Ratio
C
id HR (95% CI) pvalue
AGPAT1 1.603(1.063−2.416) 0.024
AGPAT2 1.041(0.824−1.314) 0.738
AGPAT3 0.712(0.461−1.099) 0.125
AGPAT4 1.124(0.818−1.544) 0.470
DGAT2 1.626(1.197−2.211) 0.002
DGKA 0.862(0.604−1.23) 0.413
DGKB 2.584(0.896−7.454) 0.079
DGKD 1.658(1.056−2.603) 0.028
DGKE 0.451(0.268−0.759) 0.003
DGKG 3.311(1.799−6.093) 0.000
DGKH 0.983(0.61−1.582) 0.943
DGKQ 0.84(0.548−1.287) 0.424
DGKZ 1.014(0.636−1.616) 0.953
GK 1.257(0.783−2.021) 0.344
GPAM 1.253(0.829−1.894) 0.284
GPAT2 1.237(0.865−1.77) 0.244
GPAT3 1.255(0.964−1.634) 0.092
GPAT4 1.415(0.961−2.085) 0.079
LCLAT1 1.739(1.119−2.702) 0.014
LIPG 1.316(1.063−1.628) 0.012
MBOAT1 0.833(0.689−1.008) 0.060
PLPP1 0.749(0.568−0.987) 0.040
PLPP2 0.727(0.637−0.83) 0.000
PLPP3 1.089(0.91−1.303) 0.350
1 2 3
D
AGPAT1 high low
Overall survival
p<0.001
Time(years)
DGAT2 high low
p=0.001
DGKD high low
p=0.021
LCLAT1 high low
p=0.013
LIPG high low
p=0.004
DGKE high low
p<0.001
DGKG high low
p<0.001
PLPP1 high low
p=0.012
PLPP2 high low
p<0.001

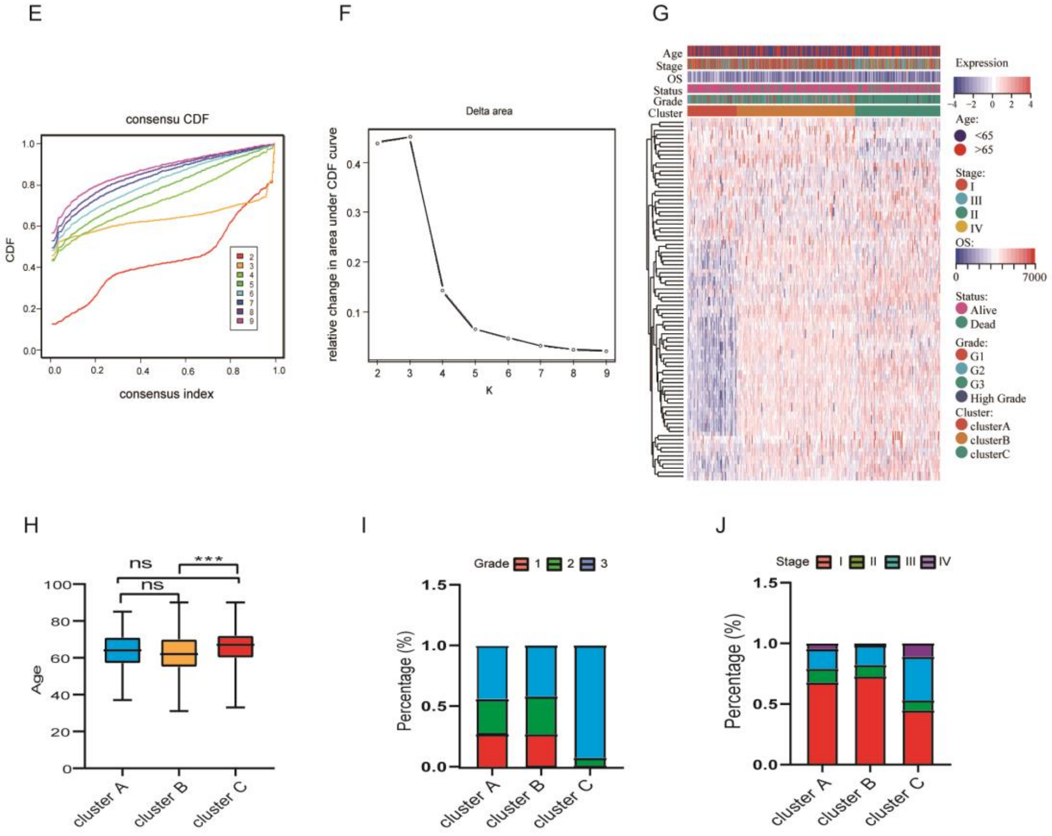

Figure S2. The univariate Cox and functional enrichment analysis of LMRGs in UCEC patients.

(**A**)-(**B**) The Kyoto encyclopedia of genes and genomes (KEGG) and Gene Ontology (GO) analysis of DE- LMRGs in UCEC. (**C**) The forest plot showed the univariate analysis of prognosis-related LMRGs. A hazard ratio>1 represents risk genes, and a Hazard ratio<1 represents protective genes. $P<0.05$ is considered significant. (**D**) K-M analyses of 9 significant genes showed that high expression of AGPAT1, DGAT2, DGKD, DGKG, LCLAT1 and LIPG had a bad prognosis. DGKE, PLPP1 and PLPP2 was favorable genes, with prolonged survival after high expression. (**E**)-(**F**) Consensus clustering cumulative distribution function (CDF) and relative change in area under CDF curvefor k = 2 to 9, which were utilized to indicate the stability of the cluster. (**G**) Heatmap of the expression distribution of 88 DE- LMRGs based on three clusters. (**H**)-(**J**) The clinical characteristics (Age, grade and stage) of the three clusters. *$P<0.05$, **$P<0.01$, ***$P<0.001$

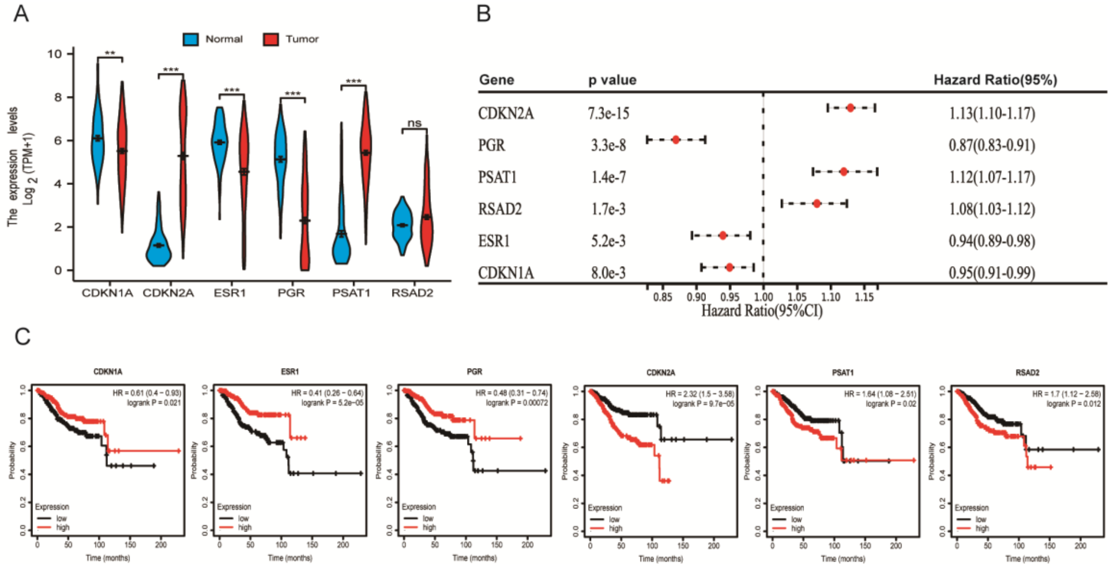

Figure S3. Lasso Cox regression analysis

(**A**) The expression level of 6 risk genes in UCEC patients of TCGA. (**B**)The univariate Cox of 6 risk genes (p<0.05). (**C**)The K- M survival analysis of six risk genes in UECE patients.

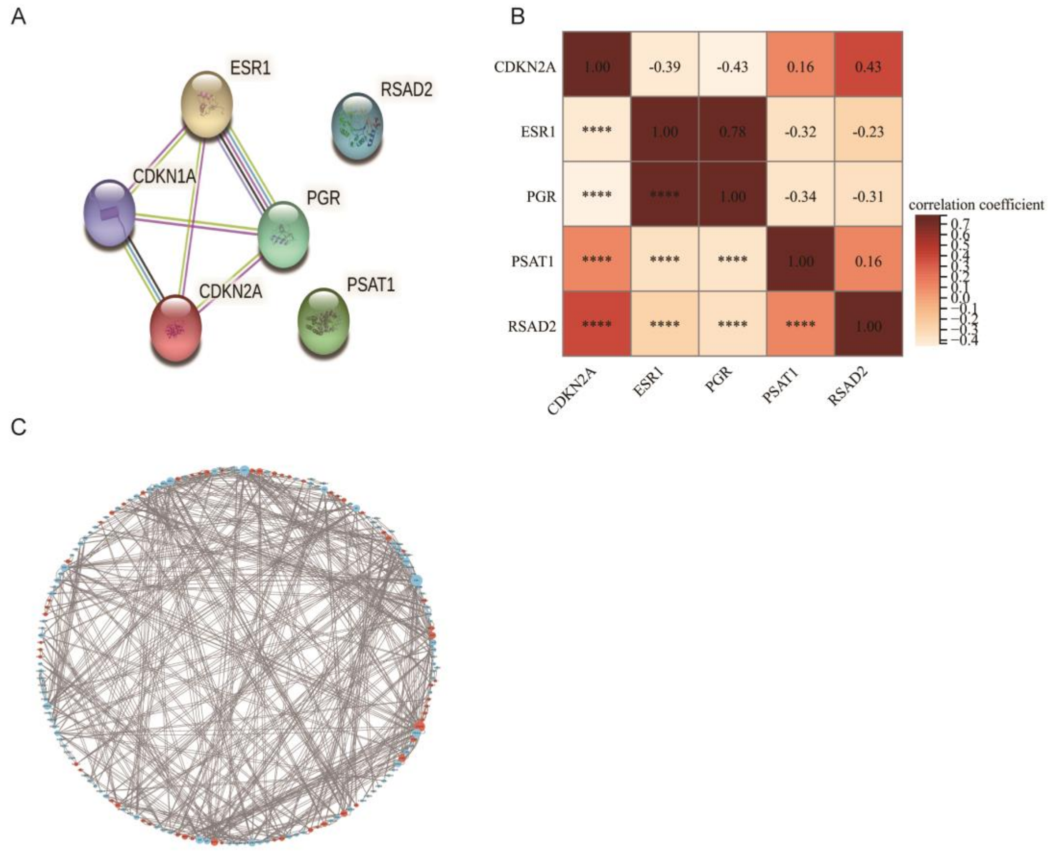

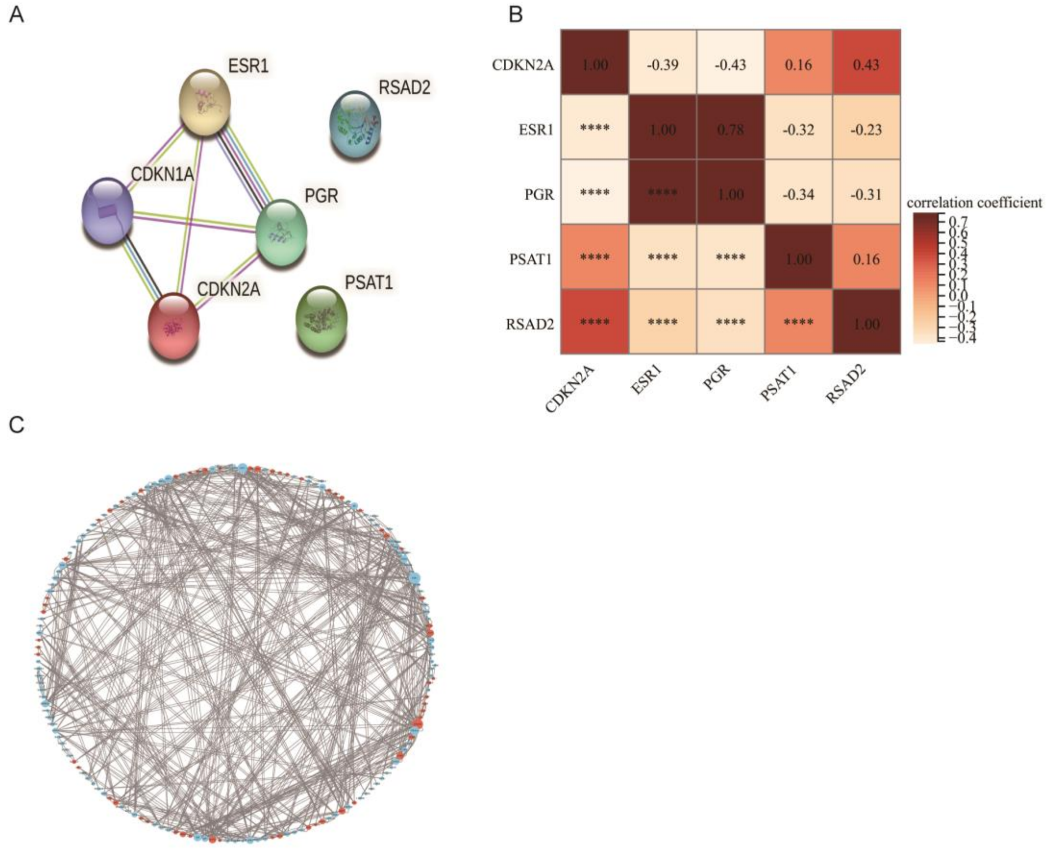

Figure S4. The network of signature.

(**A**) The protein-protein interaction (PPI) network of six risk genes. (**B**) The relationship analysis between the risk genes. (**C**) The PPI network of different genes based on the signature.

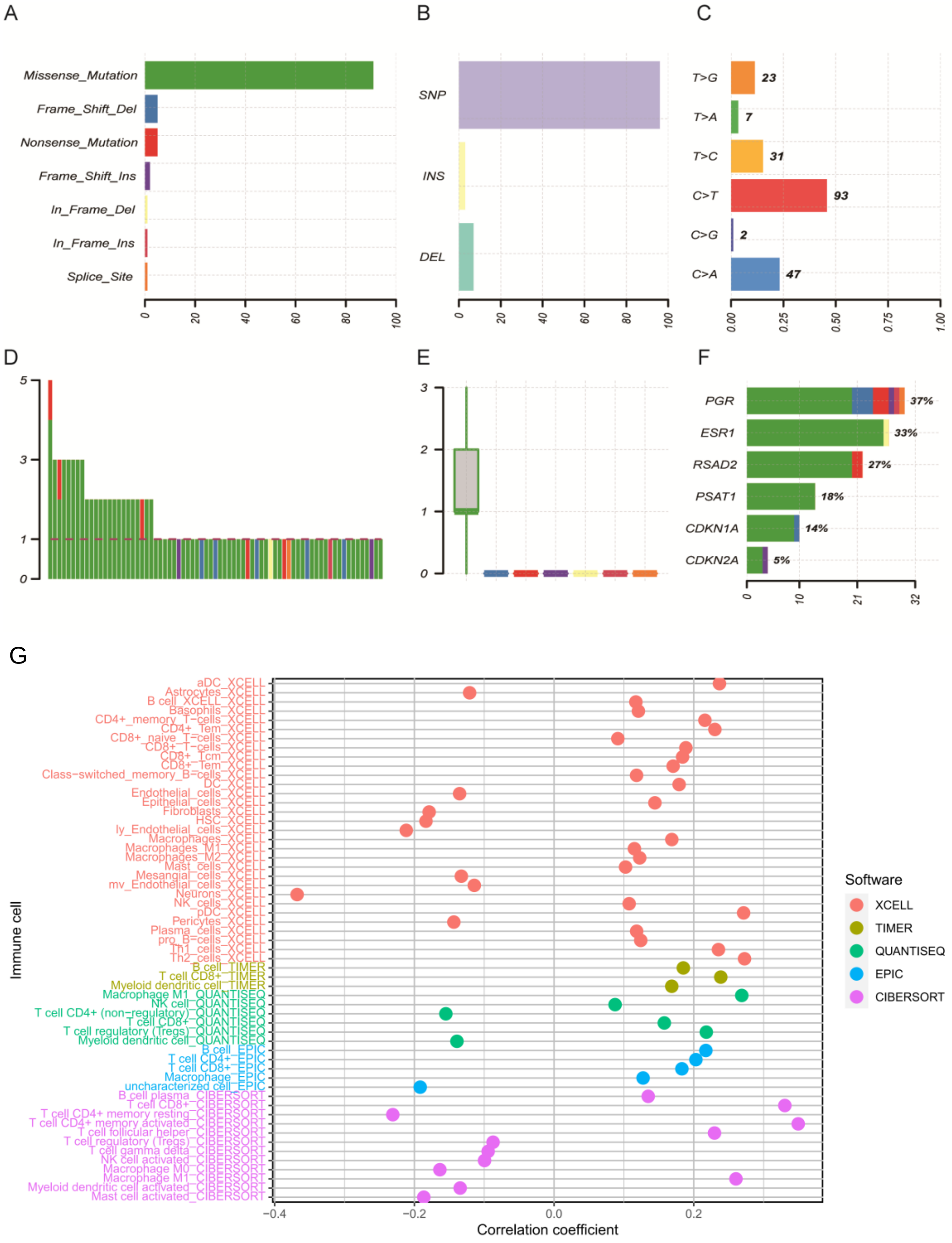
A
B
C
Missense_Mutation
Frame_Shift_Del
Nonsense_Mutation
Frame_Shift_Ins
In_Frame_Del
In_Frame_Ins
Splice_Site
SNP
INS
DEL
T>G
T>A
T>C
C>T
C>G
C>A
23
7
31
93
2
47
D
E
F
PGR
ESR1
RSAD2
PSAT1
CDKN1A
CDKN2A
37%
33%
27%
18%
14%
5%
G
Immune cell
Correlation coefficient
Software
XCELL
TIMER
QUANTISEQ
EPIC
CIBERSORT

H

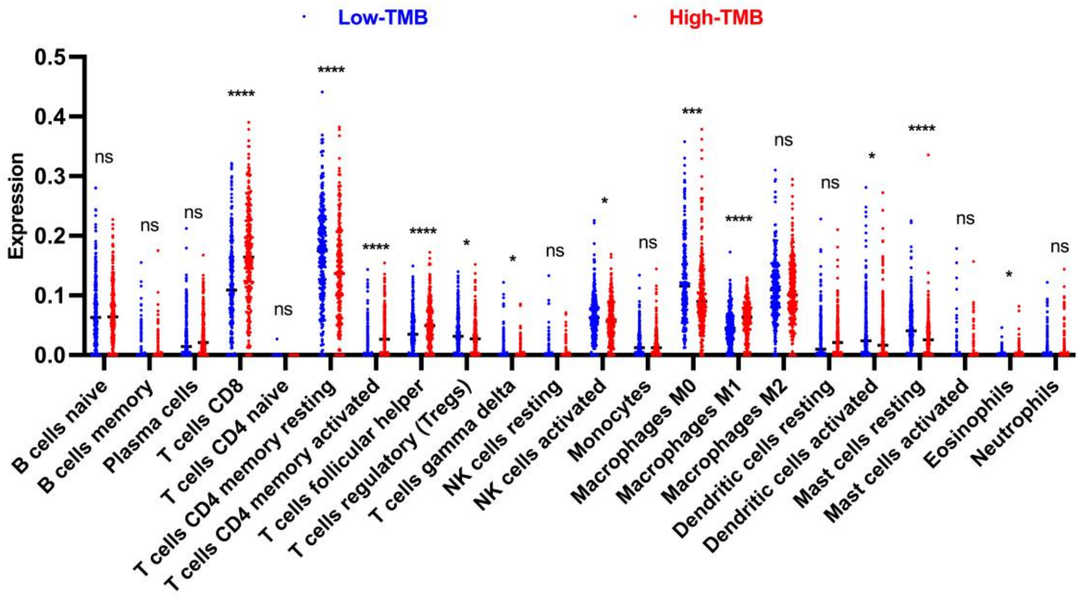

Figure S5. The detail of mutation information.

(**A**) The missense mutation accounts for the largest proportion in variant classification. (**B**) SNP occupied the most fraction in variant type. (**C**) C>T was the most frequent of SNV. (**D**)-(**E**) The number of altered bases in each sample and the mutation types in different colors. (**F**) The mutation of 6 risk genes in UCEC samples. (**G**) Bubble graphic demonstrates the correlation between TMB and immune cells. (**H**) The distribution of immune cells in high- and low-TMB group. ns $P > 0.05$, *$P<0.05$, **$P<0.01$, ***$P<0.001$.

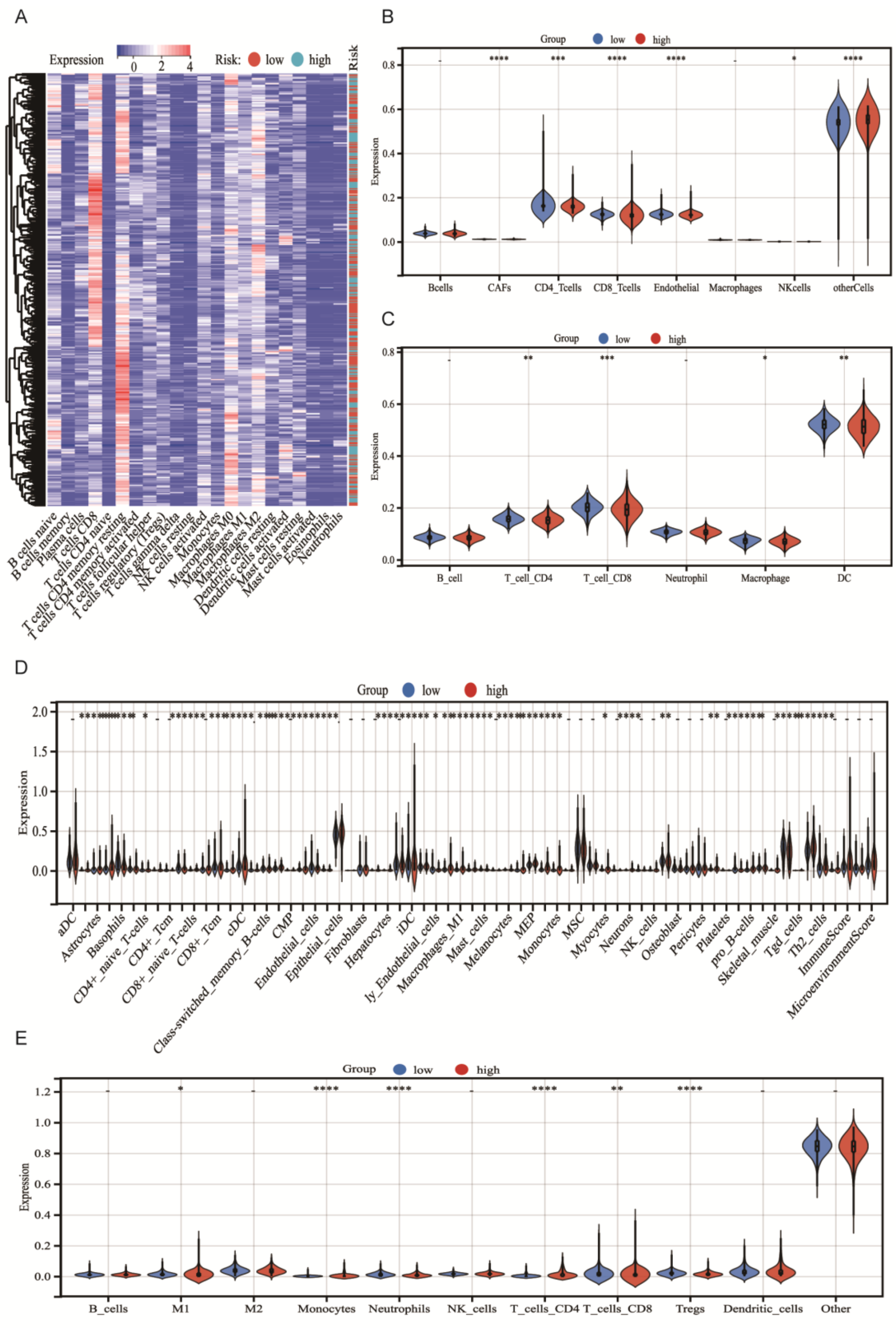

Figure S6. Immune Infiltration of LMRG- FAG- Based Signature

(**A**) Heatmap showed the fraction of 22 immune cells in each patient from the high- and low-risk groups analyzed by CIBERSORT. (**B**)-(**E**) Tumor-infiltrating immune cells analysis of patients in different risk group by EPIC, TIMER, xCELL and quanTIseq database (Wilcoxon, *P*<0.05).

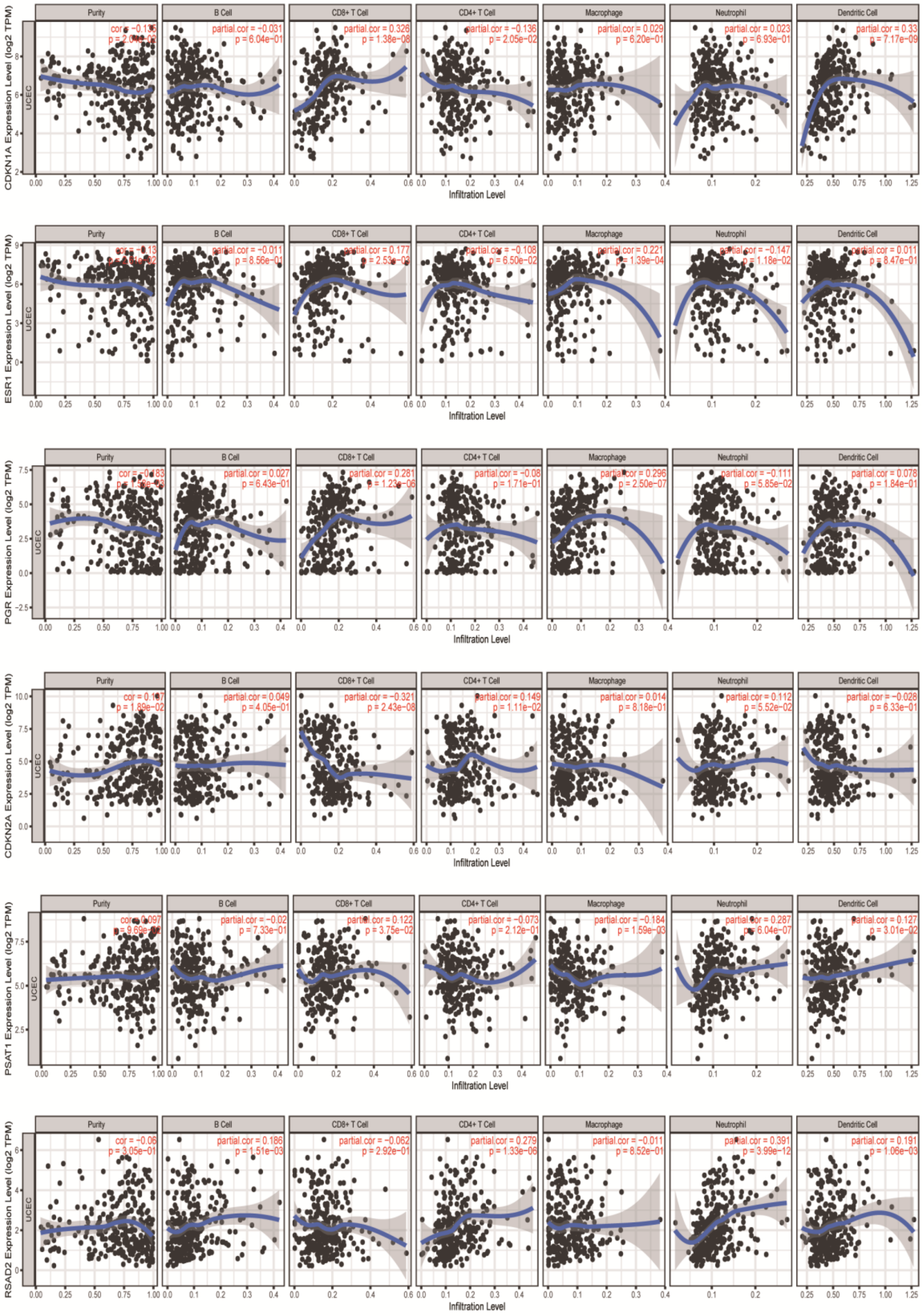

Figure S7. The relationship between the expression of six risk genes (log2 TPM) and immune infiltrating cells in UCEC patients by the TIMER database (*P*<0.05).

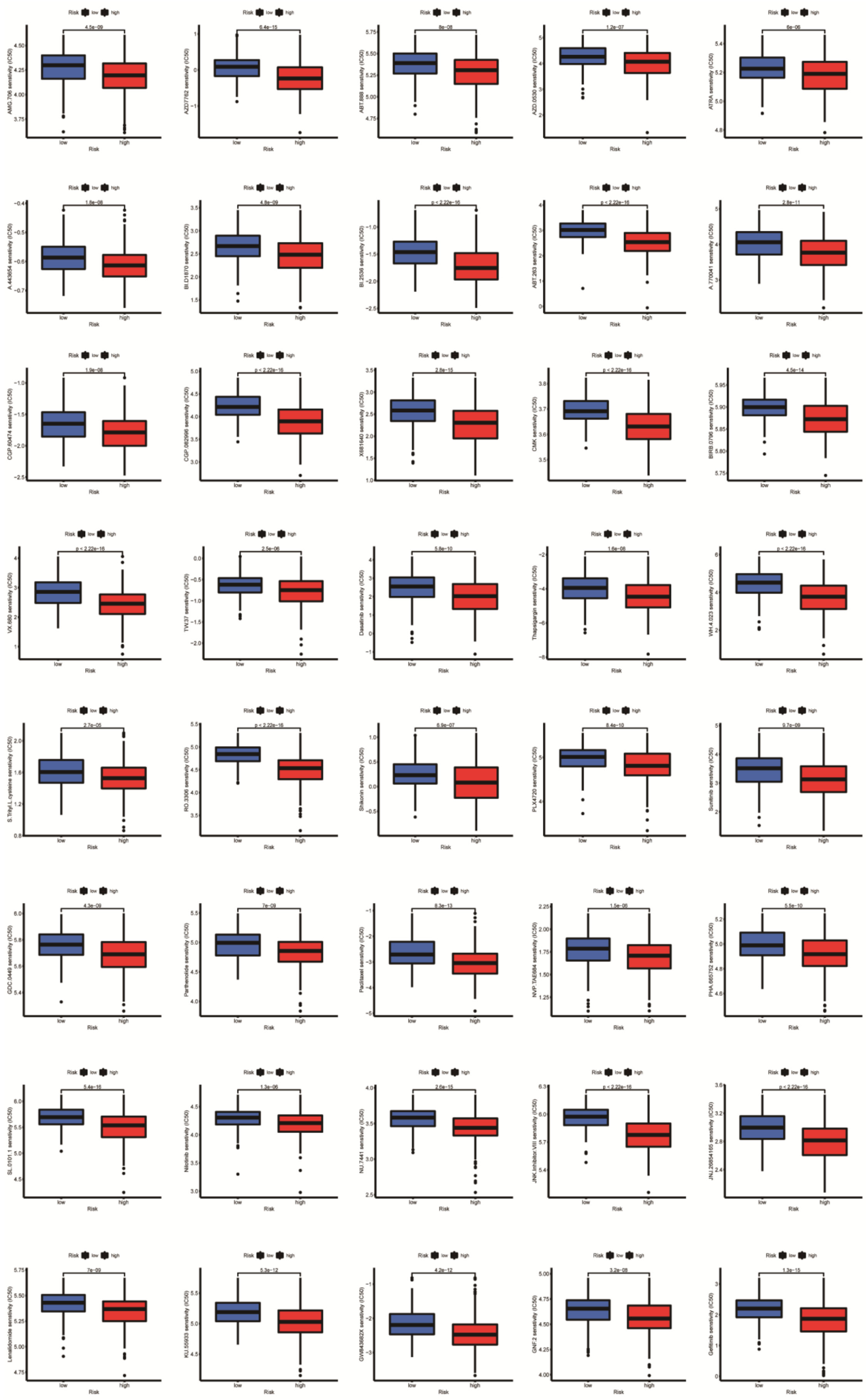

Figure S8. The half-maximal inhibitory concentration (IC50) values of potential chemotherapy agents and small molecule drugs were estimated by pRRophetic algorithm.

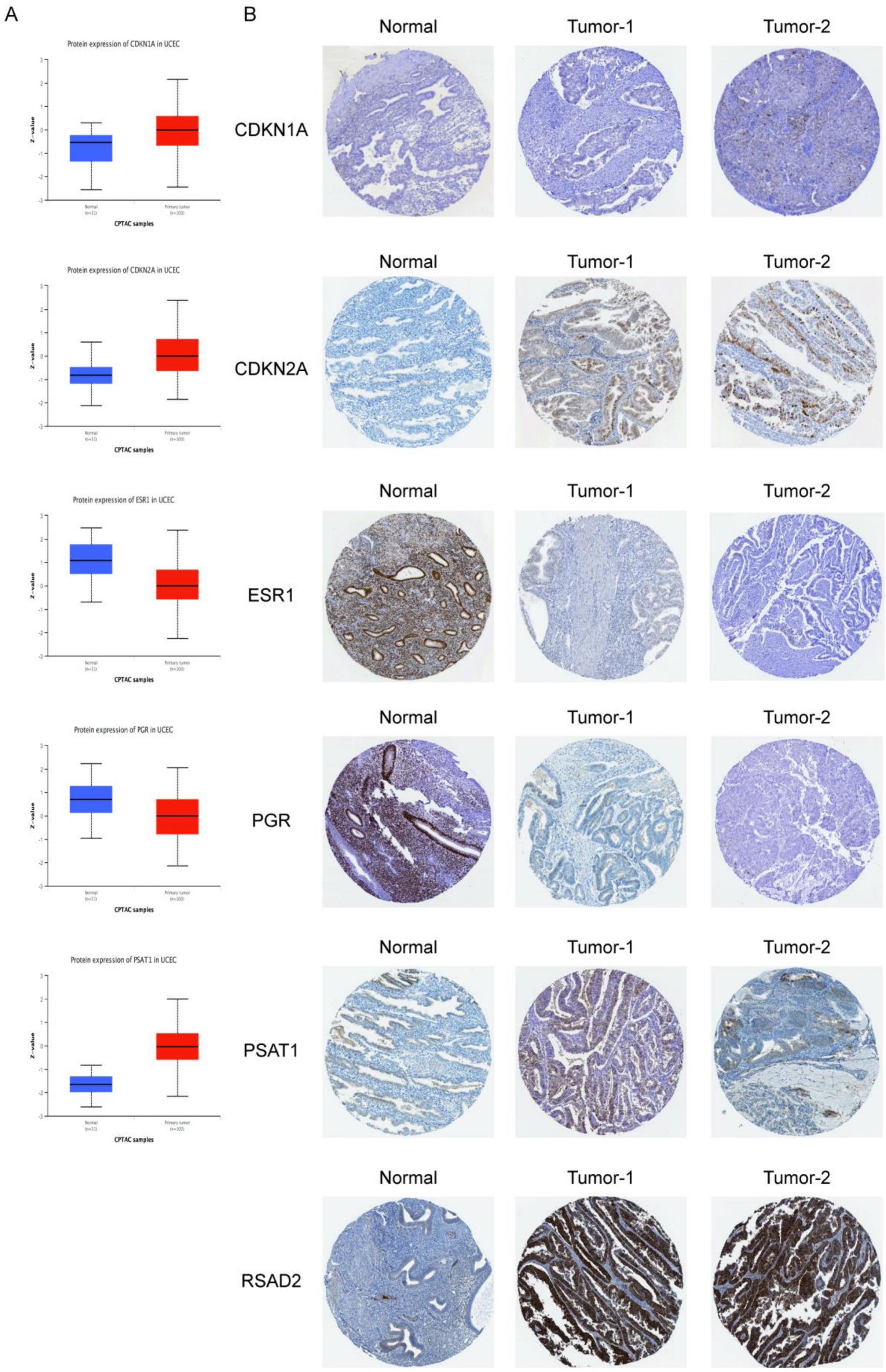

Figure S9. The protein expression of six risk genes

(**A**) Clinical Proteomic Tumor Analysis Consortium (CPTAC) database was used to demonstrate the protein expression of six risk genes (*P*<0.05, the result of RSAD2 was missing). (**B**) The expression of six risk genes between normal and UCEC tumor tissues was shown by HPA (Human Protein Atlas) database.
