# Supplementary material for: Comprehensive Analysis of Prognosis and Immune Landscapes Based on Lipid-Metabolism- and Ferroptosis-Associated Signature in Uterine Corpus Endometrial Carcinoma
Source: Diagnostics (Basel). 2023 Feb 24;13(5):870. doi: 10.3390/diagnostics13050870 (PMC10000778; doi:10.3390/diagnostics13050870)
Supplement: Supplementary file 1 [file diagnostics-13-00870-s001.zip › Supplementary Tables.pdf]

Supplementary Tables

Table S1. Characteristics of patients with UCEC in the TCGA

**Characteristics**	No.(%)
**Age**	
≥ 65 years	308(56.6)
< 65 years	236(43.4)
**Survival Status**	
Alive	457(84.0)
Dead	87(16.0)
**Stage**	
**I**	340 (62.5)
II	52(9.6)
III	123(22.6)
IV	29(5.3)
**Grade**	
G1	99(18.2)
G2	122(22.4)
G3	312(57.4)
High Grade	11(2.0)

Table S2. The detailed lipid metabolism-related gene sets from the MsigDB and KEGG database.

Database	Gene-set
GSEA	KEGG_GLYCEROLIPID_METABOLISM<br>KEGG_GLYCEROPHOSPHOLIPID_METABOLISM<br>KEGG_SPHINGOLIPID_METABOLISM<br>KEGG_FATTY_ACID_METABOLISM<br>KEGG_STEROID_BIOSYNTHESIS<br>KEGG_STEROID_HORMONE_BIOSYNTHESIS<br>KEGG_ETHER_LIPID_METABOLISM<br>KEGG_GLYCOSPHINGOLIPID_BIOSYNTHESIS_LACTO_AND_<br>NEOLACTO_SERIES<br>KEGG_GLYCOSPHINGOLIPID_BIOSYNTHESIS_GLOBO_SERIES<br>KEGG_GLYCOSPHINGOLIPID_BIOSYNTHESIS_GANGLIO_SERIES
HALLMARK	HALLMARK_CHOLESTEROL_HOMEOSTASIS<br>HALLMARK_ADIPOGENESIS<br>HALLMARK_FATTY_ACID_METABOLISM
REACTOME	REACTOME_METABOLISM_OF_LIPIDS<br>REACTOME_PHOSPHOLIPID_METABOLISM<br>REACTOME_GLYCOSPHINGOLIPID_METABOLISM

	REACTOME_CHOLESTEROL_BIOSYNTHESIS<br>REACTOME_FATTY_ACIDS<br>REACTOME_TRANSCRIPTIONAL_REGULATION_OF_WHITE_ADIPOCYTE_DIFFERENTIATION<br>REACTOME_REGULATION_OF_LIPID_METABOLISM_BY_PPARALPHA<br>REACTOME_SPHINGOLIPID_METABOLISM<br>REACTOME_FATTY_ACID_METABOLISM<br>REACTOME_GLYCEROPHOSPHOLIPID_BIOSYNTHESIS	
KEGG	hsa00061	Fatty acid biosynthesis
	hsa00062	Fatty acid elongation
	hsa00071	Fatty acid degradation
	hsa00072	Synthesis and degradation of ketone bodies
	hsa00100	Steroid biosynthesis
	hsa00120	Primary bile acid biosynthesis
	hsa00140	Steroid hormone biosynthesis
	hsa00561	Glycerolipid metabolism
	hsa00564	Glycerophospholipid metabolism
	hsa00565	Ether lipid metabolism
	hsa00590	Arachidonic acid metabolism
	hsa00591	Linoleic acid metabolism
	hsa00592	alpha-Linolenic acid metabolism
	hsa00600	Sphingolipid metabolism
	hsa01040	Biosynthesis of unsaturated fatty acids
	hsa04979	Cholesterol metabolism
	hsa00260	Glycine, serine and threonine metabolism
	hsa04146	Peroxisome
	hsa04925	Cholinergic sypse
	hsa04923	Regulation of lipolysis in adipocytes
	hsa04072	Phospholipase D sigling pathway
		Sum lipid metabolism-related gene： 1457

Table S3. The list of Lipid Metabolism-Related Genes

The list of Lipid Metabolism-Related Genes								
1	MBOAT2	UGT1A9	JAGN1	SMS	GBA2	PLEKHA4	PEDS1	IDH2
2	GPAM	UGT1A8	EPHX2	CBR3	GC	PLEKHA5	EGFR	ATP2B1
3	DGKZ	UGT1A7	IDH3G	NTHL1	GDE1	PLEKHA6	PEX7	PAOX
4	DGKE	UGT1A6	GPX3	AADAT	GDPD1	PLEKHA	ADCY1	ATP2B3

						8		
5	DGKD	UGT1A5	ELMOD3	HSPH1	GDPD3	PLIN1	PLCB4	ATP2B4
6	DGKH	UGT1A4	ORM1	APEX1	GDPD5	PLIN3	BPGM	ATP2B2
7	MBOAT1	UGT1A3	RETSAT	ACSS1	GGPS1	PLPP6	PEDS1-UBE2V1	HAO1
8	AGPAT3	UGT1A10	ESRRA	ENO3	GGT1	PNPLA2	PDGFRA	RAF1
9	AGPAT4	UGT1A1	HIBCH	LGALS1	GGT5	PNPLA4	PEX5	MAP2K1
10	DGKQ	SULT2B1	SUCLG1	METAP1	GK3P	PNPLA5	ADCY2	PRDX5
11	DGKB	SULT1E1	STAT5A	ACSM3	GLB1L	PNPLA6	ITPR1	MC2R
12	DGKG	STS	ITGA7	D2HGDH	GLIPR1	PNPLA7	LPA	MAP2K2
13	DGKA	SRD5A3	MRAP	ADSL	GLTP	PNPLA8	PHGDH	MAPK1
14	PLPP3	SRD5A2	PLIN2	SUCLA2	GM2A	POMC	PDGFRB	SOD2
15	DGKI	SRD5A1	CYC1	SDHA	GPCPD1	PON1	PEX5L	MAPK3
16	LCLAT1	HSD3B2	RNF11	XIST	GPS2	PON2	ADCY3	NOS2
17	GPAT3	HSD3B1	ALDOA	OSTC	GPX1	PON3	ITPR2	PRDX1
18	GPAT4	HSD17B8	SULT1A1	GLUL	GPX2	PPARD	PSAT1	GSTK1
19	PLPP1	HSD17B6	DDT	GABARAPL1	GRHL1	PPARGC1A	KIT	XDH
20	PLPP2	HSD17B3	SDHB	AQP7	GSTM4	PPARGC1B	ADCY4	DHRS4
21	AGPAT1	HSD17B2	CD151	PRDX6	HACD1	PPM1L	ITPR3	NR4A2
22	AGPAT2	HSD17B12	SLC27A1	ERP29	HACD2	PPP1CA	PSPH	NR4A1
23	GPAT2	HSD17B1	BCKDHA	H2AZ1	HACD3	PPP1CB	INSR	F2
24	LIPG	HSD11B2	C3	GAPDHS	HACD4	PPP1CC	PEX13	AVP
25	GK	HSD11B1	LEP	GAD2	HACL1	PPT1	ADCY5	CXCL8
26	DGAT2	CYP7B1	ADCY6	PTPRG	HDAC3	PPT2	PRKCA	PDE2A
27	GK2	CYP7A1	ELOVL6	IL4I1	HELZ2	PRKAA2	NCEH1	F2R
28	GLA	CYP3A7	LTC4S	TP53INP2	HILPDA	PRKAB2	GCAT	GRM1
29	ALDH7A1	CYP3A5	SPARCL1	PDHA1	HMGCLL1	PRKACA	GRB2	GRM2
30	AGK	CYP3A43	RMDN3	RAP1GDS1	HPGDS	PRKACB	PEX12	GRM3
31	TKFC	CYP3A4	MTCH2	BLVRA	HSD17B13	PRKACG	PRKCB	GRM4
32	AWAT2	CYP21A2	SOWAHC	SERINC1	HSD17B14	PRKAG2	GAB1	GRM5
33	AKR1B1	CYP1B1	SLC1A5	BMPR1B	HSD3B7	PRKD1	PEX10	GRM6
34	PNLIPRP2	CYP1A1	CMPK1	RDH11	HTD2	PRKD2	ADCY7	GRM7
35	PNLIPRP1	CYP19A1	REEP6	ENO2	IDI2	PRKD3	PRKCG	GRM8
36	PNLIP	CYP17A1	NDUFA5	AACS	INPP4A	PRXL2B	ALAS2	AVPR1B
37	DGAT1	CYP11B2	FZD4	ABCB11	INPP4B	PSAP	GAB2	AVPR2

38	GLYCTK	CYP11B1	DRAM2	ABCB4	INPP5D	PTEN	PEX2	LPAR1
39	LPL	CYP11A1	MGST3	ABCC1	INPP5E	PTGDS	ADCY8	LPAR2
40	ALDH1B1	COMT	ATP1B3	ABCC3	INPP5F	PTGES	PRKCE	LPAR3
41	ALDH2	AKR1D1	RETN	ABCD1	INPP5J	PTGES2	APOC1	LPAR4
42	AKR1A1	AKR1C4	STOM	ABHD3	INPP5K	PTGES3	MAOB	LPAR5
43	LIPC	AKR1C3	ESYT1	ABHD4	INPPL1	PTGIS	SHC1	LPAR6
44	MGLL	AKR1C2	GHITM	ABHD5	INSIG1	PTGR1	PXMP2	PTGFR
45	ALDH9A1	AKR1C1	DNAJC1<br>5	ACACA	INSIG2	PTGR2	ADCY9	CXCR1
46	ALDH3A2	AGPS	GADD45<br>A	ACACB	KPNB1	PTGS1	APOC2	CXCR2
47	PNPLA3	ENPP2	VEGFB	ACAD10	LBR	PTGS2	SHC2	G12
48	CEL	ENPP6	PFKL	ACAD11	LHB	PTPMT1	MPV17	PTK2B
49	LIPF	PAFAH1B1	COQ3	ACBD4	LIPH	PTPN13	APOH	G13
50	PEMT	PAFAH1B2	NABP1	ACBD5	LIPI	RAB14		CYTH3
51	PLA2G15	PAFAH1B3	CYP4B1	ACBD6	LPIN1	RAB4A	HSD11B1L	CYTH4
52	GPD1L	PAFAH2	PPM1B	ACBD7	LPIN2	RAB5A	SHC3	CYTH2
53	LPGAT1	PLA2G7	ARAF	ACOT1	LPIN3	RAN	MPV17L2	CYTH1
54	MBOAT7	B3GNT4	CAVIN1	ACOT11	LRP2	RGL1	ANGPTL3	RAPGEF3
55	PCYT1A	B3GNT2	COL4A1	ACOT12	LTA4H	RORA	AOC2	RAPGEF4
56	PLA2G2A	ST3GAL6	IMMT	ACOT13	MAPKAPK<br>2	RUFY1	SHC4	DNM1
57	PLA2G4A	B4GALT1	DHRS7	ACOT4	MBTPS1	RXRA	MPV17L	DNM3
58	PLA2G5	B3GNT5	COL15A<br>1	ACOT6	MBTPS2	RXRB	CREB1	DNM2
59	PLA2G4B	FUT9	NMT1	ACOT7	MCAT	SACM1L	PTPN11	UGT2B11
60	PTDSS2	FUT7	COQ5	ACOT9	MECR	SAMD8	PXMP4	UGT2B10
61	PCYT2	FUT6	LAMA4	ACOX2	MED1	SAR1B	NPPA	UGT2A3
62	CDS1	FUT5	BAZ2A	ACOXL	MED10	SBF1	ATF2	UGT2A1
63	PGS1	FUT4	IDH3A	ACP6	MED11	SBF2	ANGPTL8	FAH
64	CDIPT	FUT3	LIFR	ACSBG1	MED12	SCAP	AMT	UBE2L6
65	CDS2	FUT2	PREB	ACSBG2	MED13	SCD5	SOS1	FHL2
66	GPD1	FUT1	PTGER3	ACSF2	MED13L	SEC23A	NPR1	PLD6
67	CHAT	B4GAT1	GPHN	ACSF3	MED14	SEC24A	ATF4	PLCB3
68	LPCAT1	GCNT2	PFKFB3	ACSM6	MED15	SEC24B	SOS2	CAMK2B
69	PTDSS1	B3GNT3	GPX4	ACSS3	MED16	SEC24C	PEX11B	ACLY
70	ACHE	B3GALT5	SSPN	AGMO	MED17	SEC24D	PRKG1	MIF
71	PLA2G3	ABO	SQOR	AGPAT5	MED18	SELENOI	CREB3	FIG4
72	ETNK1	B4GALT3	MTARC<br>2	AGT	MED19	SERPINA<br>6	SORT1	PLEKHA<br>1
73	JMJD7-PL<br>A2G4B	B4GALT2	DLD	AHR	MED20	SIN3A	GCSH	CETP
74	CHPT1	ST3GAL4	ITIH5	AHRR	MED21	SIN3B	HRAS	DDO

75	PLA2G6	B4GALT4	CD302	AKR1B15	MED22	SLC10A1	PEX11G	IFNGR1
76	PLA2G2E	ST3GAL3	ATL2	ALAS1	MED23	SLC10A2	PRKG2	SUCLG2
77	PLA2G10	B3GALT2	TKT	ALB	MED24	SLC25A17	CREB3L1	FITM1
78	LPCAT3	B3GALT1	UQCRC1	ALDH3B1	MED25	SLC25A20	APOB	PLEKHA2
79	ETNK2	ST8SIA1	CAT	ALDH3B2	MED26	SLC27A2	DAO	PGAM4
80	PLA2G12B	A4GALT	OMD	ALOX12	MED27	SLC27A3	PLPP5	CAMK2G
81	PLA2G2F	ST3GAL2	DLAT	ALOX12B	MED28	SLC27A5	KRAS	SLC5A6
82	LYPLA1	GBGT1	MRPL15	ALOX15	MED29	SLC44A1	CREB3L2	CPOX
83	PLA2G1B	ST3GAL1	RIOK3	ALOX15B	MED30	SLC44A2	PCSK9	FITM2
84	ADPRM	HEXB	RTN3	ALOX5	MED31	SLC44A3	AGXT2	PLEKHA3
85	PHOSPHO1	NAGA	CHUK	ALOX5AP	MED4	SLC44A4	PLPP4	AKR1B10
86	TAFAZZIN	B3GALNT1	G3BP2	ALOXE3	MED6	SLC44A5	NRAS	CAMK4
87	GNPAT	HEXA	SDHC	ALPI	MED7	SLC51A	CREB3L3	UGT2B4
88	LPCAT4	ST6GALNAC4	SAMM50	AMACR	MED8	SLC51B	LDLRAP1	UGT2B28
89	PLA2G4E	ST6GALNAC3	ARL4A	ANKRD1	MED9	SLCO1A2	GATM	UGT2B17
90	CHKA	ST6GALNAC6	SNCG	APOA1	MFSD2A	SLCO1B1	MRAS	UGT2B15
91	LYPLA2	B4GALNT1	PDCD4	APOA2	MID1IP1	SLCO1B3	CREB3L4	UQCR10
92	PLA2G2C	ST6GALNAC5	COQ9	APOA5	MIGA1	SMARCD3	MYLIP	FMO1
93	PLA2G2D	SLC33A1	APLP2	ARF1	MMAA	SP1	GAMT	FAR2
94	LPCAT2	ST3GAL5	SOD1	ARF3	MMUT	SPNS2	RRAS	PLB1
95	PLA2G12A	B3GALT4	PTCD3	ARNT	MOGAT1	SPTLC3	CREB5	PGAM2
96	PLD1	ST8SIA5	PHLDB1	ARNT2	MOGAT2	SPTSSA	CHDH	LRTOMT
97	CHKB	FDPS	HSPB8	ARNTL	MOGAT3	SPTSSB	RRAS2	TANK
98	PISD	IDI1	AIFM1	ARSB	MORC2	SREBF1	ATF6B	AOC3
99	PLD2	HMGCS1	CCNG2	ARSD	MTF1	STAR	LRP1	FDX1
100	PCYT1B	MVD	PPP1R15B	ARSF	MTM1	STARD10	DHRS11	PLBD1
101	LCAT	LDLR	MDH2	ARSG	MTMR1	STARD3	RALGDS	INS
102	CRLS1	ALDOC	ABCA1	ARSH	MTMR10	STARD3NL	ATF1	CAMK1
103	GPD2	SCD	COX7B	ARSI	MTMR12	STARD5	BHMT	ANGPTL4
104	GAL3ST1	PMVK	MYLK	ARSJ	MTMR14	STARD6	RALA	UROD
105	SGPP2	MVK	COX8A	ARSK	MTMR2	STARD7	DAGLA	FDX2
106	GLB1	FADS2	DHRS7B	ARSL	MTMR3	SULT2A1	LRPAP1	PLD3

107	GALC	HMGCR	MIGA2	ARV1	MTMR4	SUMF1	DMGDH	PEX26
108	SGMS2	ANXA13	ITSN1	AWAT1	MTMR6	SUMF2	RALB	CAMK2A
109	GBA	SREBF2	RREB1	BAAT	MTMR7	SUMO2	AQP7B	ACO2
110	SPHK2	ACSS2	CMBL	BCHE	MTMR8	SYNJ1	DAGLB	KMT5A
111	NEU2	ATF3	UBC	BDH1	MTMR9	SYNJ2	PIPOX	FDXR
112	NEU1	ETHE1	ATP5PO	BDH2	NCOA1	TBL1X	KCNK3	PLD4
113	ACER2	ECH1	PRDX3	BMX	NCOA2	TBL1XR1	SARDH	GS
114	UGCG	CBS	DBT	CARM1	NCOA3	TBXAS1	KCNK9	CAMK2D
115	DEGS2	GUSB	NDUFS3	CAV1	NCOA6	TECR	VDAC1	TM7SF2
116	ARSA	FASN	NKIRAS1	CBR4	NCOR1	TECRL	CYP3A7-CYP3A51P	NSDHL
117	SPHK1	LGALS3	RAB34	CCNC	NCOR2	TGS1	GNMT	SOAT2
118	SGPL1	ATF5	CIDEA	CDK19	NFYA	THEM4	IRS1	UGT2B7
119	NEU3	ANXA5	UQCRQ	CDK8	NFYB	THEM5	KCNJ5	SCARB1
120	SMPD4	TP53INP1	PEX14	CEPT1	NFYC	THRAP3	VDAC2	CHCHD10
121	SGMS1	GSTM2	BCL6	CERS1	NPAS2	THRSP	DGKK	AK2
122	ACER3	AVPR1A	COX6A1	CERS2	NR1D1	TIAM2	IRS2	APOE
123	CERK	PLSCR1	DNAJB9	CERS3	NR1H2	TMEM86B	ATP1A1	CA6
124	SMPD2	CLU	MAP4K3	CERS4	NR1H3	TNFAIP8	VDAC3	HCCS
125	ENPP7	ERRFI1	ANGPT1	CERS5	NR1H4	TNFAIP8L1	IRS4	EPHX1
126	DEGS1	TRIB3	UBQLN1	CERS6	NRF1	TNFAIP8L2	ATP1A2	DLST
127	SGPP1	CXCL16	NDUFB7	CERT1	NUDT19	TNFAIP8L3	CTH	FABP9
128	NEU4	TNFRSF12A	SLC19A1	CGA	NUDT7	TNFRSF21	ATP1A3	FADS1
129	ACER1	ACTG1	ABCB8	CH25H	OCRL	TPTE	SDS	FAM120B
130	ASAH1	JAG1	SLC66A3	CHD9	OLAH	TPTE2	B4GALT5	FAR1
131	SMPD1	LGMN	POR	CIDEC	ORMDL1	TSPO	AKT1	PLAAT2
132	B4GALT6	FBXO6	UCP2	CLOCK	ORMDL2	TSPOAP1	ATP1A4	PLAAT3
133	SPTLC2	GPX8	UQCR11	CPNE1	ORMDL3	TXNRD1	SDSL	PLAAT4
134	SPTLC1	PNRC1	ACOT8	CPNE3	OSBP	UBE2I	AKT2	PLAAT5
135	SMPD3	ANTXR2	MIX23	CPNE6	OSBPL10	VAC14	ABCD2	PEX6
136	KDSR	MAL2	ELOVL5	CPNE7	OSBPL1A	VAPA	ATP1B4	ADRB3
137	UGT8	CD9	FABP1	CPTP	OSBPL2	VAPB	SRR	PLCB2
138	ASAH2	PPARG	FABP2	CREBBP	OSBPL3	VDR	AKT3	PLTP
139	CPT1A	GLDC	HMGCL	CROT	OSBPL5	ZNF638	ABCD4	CALML4
140	CPT1C	STX5	HSD17B11	CSNK1G2	OSBPL6	KLF5	ATP1B1	UGT2A2

141	ACADS	STARD4	MLYCD	CSNK2A1	OSBPL7	TGFB1	NPC1	CAMK1D
142	ACADSB	CTNNB1	PCBD1	CSNK2A2	OSBPL8	NFKB1	TSC1	CAMK1G
143	ACADL	TMEM97	S100A10	CSNK2B	OSBPL9	CCND3	ATP1B2	FDFT1
144	ACADM	NIBAN1	VNN1	CTSA	OXCT1	EGR2	GPX6	SC5D
145	CYP4A11	PDK3	CA2	CUBN	OXCT2	PCK1	TSC2	LIPA
146	ACAT2	PLAUR	HSD17B10	CYP1A2	PCCA	WNT1	NPC2	CRAT
147	ACADVL	SEMA3B	ALDH1A1	CYP24A1	PCCB	CDK4	GPX7	NCAPH2
148	ACAT1	GNAI1	HSD17B4	CYP27A1	PCTP	KLF4	RHEB	FABP3
149	ACAA2	ABCA2	CA4	CYP2C19	PDCD6-AHRR	ADIRF	CAC1G	PLA2G4F
150	HADH	ATXN2	PTS	CYP2C8	PECR	EBF1	MTOR	OXSM
151	HADHB	NFIL3	MAOA	CYP2C9	PEX11A	WNT10B	CAC1H	CALML6
152	HADHA	ALCAM	HAO2	CYP2D6	PI4K2A	CEBPB	RHOA	GBE1
153	CYP4A22	FABP5	MCEE	CYP2E1	PI4K2B	RELA	CAC1I	BPHL
154	ADH7	S100A11	AUH	CYP2J2	PI4KA	ZNF467	PDE3B	FABP6
155	ADH6	CPEB2	HPGD	CYP2R1	PI4KB	SLC2A4	CAC1C	PLA2R1
156	ACSL6	FABP4	FH	CYP2U1	PIAS4	NR2F2	ABCG5	PGAM1
157	ADH1B	ADIPOQ	HMGCS2	CYP39A1	PIK3C2A	TNF	GPX5	GI2
158	ADH1C	LIPE	ALAD	CYP46A1	PIK3C2B	CEBPD	ARF6	CAVIN2
159	ADH4	CD36	CBR1	CYP4F11	PIK3C2G	CEBPA	CAC1D	ODC1
160	ECHS1	ADIPOR2	GRHPR	CYP4F2	PIK3C3	CYP4F12	ABCG8	FABP7
161	ADH5	ETFB	ACOT2	CYP4F22	PIK3CA	CYP2B6	PLCG1	PLAAT1
162	ACSL5	IDH1	G0S2	CYP4F3	PIK3CB	CYP2F1	CAC1F	KITLG
163	ADH1A	SORBS1	HSP90AA1	CYP4F8	PIK3CD	CYP2A13	PLCG2	CALML5
164	EHHADH	UCK1	BCKDHB	CYP8B1	PIK3CG	CYP2A7	CAC1S	HSD17B7
165	GCDH	SCP2	UROS	DBI	PIK3R1	SHMT2	APOC3	MSMO1
166	ACOX3	DECR1	YWHAH	DDHD1	PIK3R2	EGF	IGH	TOB1
167	ACSL1	CDKN2C	LDHA	DDHD2	PIK3R3	PEX16	NUDT12	PSME1
168	ACAA1	TALDO1	CRYZ	DECR2	PIK3R4	TSHB	ADORA1	FAAH2
169	CPT2	TST	RDH16	DGAT2L6	PIK3R5	SHMT1	ORAI1	PLA2G4C
170	CPT1B	MCCC1	INMT	DPEP1	PIK3R6	PDGFA	APOA4	ADRB2
171	ACOX1	PGM1	UGDH	DPEP2	PIKFYVE	PEX3	FCER1A	SYK
172	ECI2	REEP5	GSTZ1	DPEP3	PIP4K2A	AGTR1	CALML3	GRPEL1
173	ECI1	BCL2L13	IDH3B	ELOVL1	PIP4K2B	AGXT	PNLIPRP3	PPARA
174	ACSL3	SLC25A10	MDH1	ELOVL2	PIP4K2C	PDGFB	MS4A2	FABP12
175	ACSL4	ME1	ETFDH	ELOVL3	PIP4P1	PEX19	NPY	PLA2G4D
176	SOAT1	PHYH	SDHD	ELOVL4	PIP5K1A	TSHR	CALM2	PLCB1

177	LSS	PIM3	HSDL2	ELOVL7	PIP5K1B	GQ	PSAPL1	GI3
178	SQLE	YWHAG	CD1D	EP300	PIP5K1C	PDGFC	FCER1G	DHCR24
179	EBP	NDUFAB1	ALDH3A1	ESYT2	PITPNB	ABCD3	NPY1R	SLC25A1
180	CYP51A1	ADIG	SLC22A5	ESYT3	PITPNM1	ADRB1	CALM3	NBN
181	DHCR7	QDPR	PDHB	ETNPPL	PITPNM2	G11	FYN	FAAH
182	CYP27B1	CS	TDO2	FA2H	PITPNM3	PDGFD	GI1	PLA1A
183	PEX1	CALM1						

Table S4. Univariate analysis of lipid metabolism relative genes in survival of UCEC patients

id	HR	HR.95L	HR.95H	pvalue
L1CAM	1.285	1.167	1.415	0.000
B4GALNT3	0.651	0.551	0.768	0.000
SCGB2A1	0.845	0.791	0.902	0.000
KIAA1324	0.819	0.756	0.887	0.000
PLPP2	0.727	0.637	0.830	0.000
PGR	0.735	0.645	0.838	0.000
TRPM4	0.659	0.549	0.790	0.000
ESR1	0.766	0.682	0.860	0.000
CDKN2A	1.290	1.153	1.444	0.000
UBXN10-AS1	0.644	0.530	0.783	0.000
KCNK6	0.630	0.513	0.774	0.000
GRB7	1.356	1.183	1.555	0.000
SLC47A1	0.757	0.667	0.858	0.000
SLC25A35	0.698	0.592	0.823	0.000
PNOC	1.282	1.144	1.437	0.000
SPDEF	0.840	0.775	0.910	0.000
FAM189A2	0.652	0.535	0.795	0.000
MSX1	0.857	0.797	0.920	0.000
MAL	1.190	1.096	1.292	0.000
ZDHHC1	0.604	0.476	0.767	0.000
AC026336.3	0.744	0.647	0.857	0.000
IHH	0.814	0.738	0.898	0.000
SERINC2	0.748	0.649	0.862	0.000
CLDN16	1.296	1.142	1.470	0.000
ARHGAP29	1.495	1.227	1.820	0.000
IL20RA	0.696	0.582	0.832	0.000
VIM	0.739	0.636	0.859	0.000
SLC38A1	1.470	1.214	1.780	0.000
TUBB4A	1.296	1.139	1.474	0.000
CLDN6	1.145	1.069	1.225	0.000

SST	1.133	1.063	1.206	0.000
ECEL1P2	0.599	0.461	0.777	0.000
ATP2C2	0.658	0.531	0.815	0.000
KLK6	1.200	1.093	1.318	0.000
SPATA18	0.721	0.610	0.854	0.000
MLPH	0.797	0.708	0.898	0.000
TPX2	1.555	1.232	1.962	0.000
UCHL1	1.173	1.078	1.277	0.000
ANO1	0.771	0.672	0.885	0.000
DACT2	0.790	0.696	0.897	0.000
FOXA2	0.812	0.725	0.909	0.000
SCGB1D2	0.858	0.789	0.933	0.000
AC084866.1	0.835	0.756	0.922	0.000
ASRGL1	0.804	0.713	0.908	0.000
BMPR1B-DT	0.842	0.765	0.927	0.000
LRRC26	0.726	0.608	0.868	0.000
SOX17	0.829	0.746	0.922	0.001
MLF1	1.281	1.112	1.475	0.001
LPCAT2	0.655	0.514	0.834	0.001
CAPS	0.843	0.765	0.930	0.001
CDKN1A	0.763	0.652	0.891	0.001
BEX2	1.300	1.117	1.514	0.001
TJP3	0.743	0.625	0.882	0.001
CITED4	0.779	0.674	0.900	0.001
CPVL	1.296	1.114	1.506	0.001
TFF3	0.900	0.847	0.958	0.001
FBLN1	0.826	0.737	0.924	0.001
KIF1A	1.229	1.088	1.388	0.001
SAT1	0.739	0.619	0.884	0.001
GLDC	1.243	1.092	1.415	0.001
SRARP	0.638	0.487	0.837	0.001
ANG	0.726	0.598	0.882	0.001
AC013724.1	0.690	0.549	0.866	0.001
HERC5	1.343	1.120	1.609	0.001
ACSL5	0.810	0.711	0.923	0.002
FBXO17	1.288	1.100	1.507	0.002
KLK7	1.184	1.066	1.316	0.002
PSAT1	1.312	1.107	1.555	0.002
TEKT4	0.701	0.561	0.875	0.002
CLDN1	1.220	1.077	1.382	0.002
AURKA	1.470	1.153	1.875	0.002
AL354766.2	0.734	0.603	0.893	0.002
AC025154.2	0.782	0.669	0.914	0.002

C9orf152	0.782	0.667	0.915	0.002
KLHL24	1.544	1.168	2.042	0.002
EYA2	0.867	0.791	0.950	0.002
SERPINA6	0.784	0.670	0.917	0.002
ARL4D	0.801	0.693	0.925	0.003
LRRC46	0.796	0.686	0.924	0.003
RSAD2	1.294	1.094	1.530	0.003
FOXJ1	0.837	0.744	0.940	0.003
DNAJC10	0.710	0.566	0.890	0.003
SMIM6	0.818	0.717	0.934	0.003
CDH6	1.237	1.075	1.423	0.003
KIF2C	1.443	1.130	1.843	0.003
LINC01480	0.826	0.727	0.939	0.003
APLP1	1.219	1.067	1.391	0.003
NAPSB	0.855	0.769	0.950	0.004
AQP5	0.870	0.793	0.955	0.004
DNAAF1	0.722	0.580	0.899	0.004
C1orf194	0.846	0.755	0.947	0.004
MSX2	0.846	0.755	0.948	0.004
TEKT1	0.786	0.666	0.927	0.004
UPK1B	0.869	0.789	0.957	0.004
TPD52	1.522	1.142	2.030	0.004
H19	1.135	1.041	1.239	0.004
KIRREL1	1.349	1.098	1.657	0.004
PEG10	1.169	1.049	1.302	0.005
ANXA1	0.818	0.712	0.940	0.005
HOXA5	1.185	1.053	1.333	0.005
MANSC1	0.715	0.566	0.903	0.005
NT5E	0.823	0.718	0.944	0.005
AK4	1.361	1.095	1.692	0.005
WNT7A	1.141	1.039	1.253	0.006
FAM166B	0.830	0.728	0.947	0.006
SFN	0.847	0.753	0.953	0.006
SLC43A1	0.810	0.698	0.941	0.006
PLA2G4A	0.820	0.711	0.945	0.006
CFAP73	0.788	0.665	0.934	0.006
SMC4	1.379	1.093	1.739	0.007
SERPINA4	0.743	0.600	0.921	0.007
SYT13	1.264	1.067	1.498	0.007
PIGR	0.917	0.861	0.977	0.007
FBP1	0.803	0.684	0.943	0.007
LINC00261	0.823	0.714	0.949	0.007
C4BPA	0.841	0.740	0.955	0.008

PKDCC	0.842	0.741	0.956	0.008
ZNF146	1.486	1.107	1.993	0.008
GAD1	0.789	0.661	0.941	0.008
CCNE1	1.237	1.056	1.449	0.008
DPM1	1.504	1.110	2.038	0.008
MAT2A	1.460	1.098	1.942	0.009
SFRP4	0.872	0.786	0.967	0.009
CREB3L1	0.860	0.768	0.964	0.009
GLYATL2	0.868	0.780	0.966	0.010
NAMPT	1.423	1.090	1.857	0.010
RNF183	0.798	0.673	0.947	0.010
MAGEH1	1.275	1.060	1.534	0.010
LRRC71	0.784	0.652	0.943	0.010
C9orf24	0.866	0.777	0.966	0.010
CDHR4	0.828	0.717	0.956	0.010
SELENOP	1.289	1.062	1.565	0.010
RSPH1	0.833	0.724	0.958	0.010
TESC	0.844	0.741	0.962	0.011
TNNT1	1.130	1.028	1.241	0.011
STK26	1.316	1.064	1.628	0.011
MORN5	0.844	0.740	0.962	0.011
PAM	0.808	0.686	0.953	0.011
ITM2C	1.171	1.036	1.324	0.012
TMEM101	0.876	0.790	0.971	0.012
EDN3	0.845	0.740	0.964	0.012
HGD	0.854	0.755	0.966	0.012
STAP2	0.802	0.675	0.954	0.012
SERINC5	0.770	0.627	0.945	0.012
WDR38	0.860	0.764	0.968	0.013
VWA3A	0.786	0.650	0.951	0.013
SCGB1D4	0.837	0.727	0.964	0.014
FAM92B	0.824	0.706	0.962	0.014
ANKRD44-AS1	0.836	0.725	0.965	0.014
S100A1	1.134	1.025	1.255	0.014
HENMT1	1.262	1.047	1.520	0.014
ZMYND10	0.850	0.745	0.969	0.015
ATP5F1D	0.751	0.595	0.947	0.015
OVGP1	0.869	0.775	0.974	0.015
SP3	1.537	1.084	2.179	0.016
PCSK1N	1.138	1.024	1.265	0.016
PIK3CA	1.447	1.071	1.955	0.016
ENPP3	0.823	0.703	0.965	0.016
TCEA1	1.439	1.069	1.938	0.017

C20orf85	0.909	0.840	0.983	0.017
IFIT1	1.167	1.028	1.326	0.017
SRGAP3-AS2	0.808	0.677	0.963	0.018
EGFL6	1.183	1.029	1.359	0.018
PRELID3B	1.488	1.071	2.067	0.018
CCNO	0.842	0.730	0.972	0.019
AKIRIN1	1.398	1.057	1.848	0.019
HAT1	1.534	1.072	2.196	0.019
PDCD10	1.428	1.054	1.936	0.021
SLC2A1	1.236	1.032	1.481	0.022
HMGA1	1.250	1.033	1.512	0.022
AL357093.2	0.862	0.759	0.979	0.022
FAM183A	0.882	0.792	0.982	0.022
PHF6	1.451	1.054	1.997	0.022
DEK	1.309	1.038	1.652	0.023
ROPN1L	0.866	0.765	0.981	0.024
TPPP3	0.892	0.807	0.985	0.024
RAD21	1.348	1.039	1.749	0.025
SNCG	1.143	1.017	1.283	0.025
CFAP45	0.855	0.745	0.980	0.025
NPDC1	0.842	0.723	0.979	0.025
PREPL	1.493	1.050	2.123	0.026
TUBA4B	0.863	0.759	0.982	0.026
HMGA2	1.210	1.023	1.431	0.026
MPZL2	0.799	0.655	0.974	0.027
NAA50	1.420	1.041	1.937	0.027
SSB	1.385	1.038	1.849	0.027
HSPB1	0.814	0.678	0.978	0.028
FAM91A1	1.402	1.037	1.896	0.028
LANCL1	1.452	1.041	2.026	0.028
HLTF	1.335	1.031	1.730	0.029
NUP153	1.390	1.035	1.866	0.029
SCX	1.156	1.015	1.316	0.029
SNX4	1.518	1.042	2.213	0.030
AC023300.2	0.863	0.756	0.986	0.030
ECT2	1.251	1.019	1.535	0.033
PHYHD1	0.873	0.770	0.990	0.035
ACADM	1.340	1.018	1.764	0.037
PIK3R1	0.840	0.712	0.990	0.037
SNHG25	0.863	0.750	0.993	0.039
IGF2BP2	1.133	1.006	1.277	0.040
RPL41P1	1.062	1.002	1.126	0.041
PAIP1	1.393	1.013	1.915	0.042

PPP1R15B	1.375	1.012	1.868	0.042
IGHA1	0.926	0.860	0.997	0.042
PCNP	1.397	1.010	1.931	0.043
PRKAA1	1.418	1.007	1.998	0.046
FBXO2	1.130	1.002	1.275	0.046
CYP4X1	0.856	0.734	0.998	0.047
NRAS	1.370	1.004	1.870	0.047
TMPRSS13	0.839	0.704	0.999	0.049
GASK1B	0.820	0.673	0.999	0.049
